# A qualitative assessment of the sexual-health education, training and service needs of young adults in Tehran

**DOI:** 10.1186/s12889-021-11371-x

**Published:** 2021-07-13

**Authors:** Narges Sheikhansari, Charles Abraham, Sarah Denford, Mehrdad Eftekhar

**Affiliations:** 1 Medical School, University of Exeter, Exeter, UK; 2 School of Psychology, Deakin University, Melbourne, Australia; 3 Research Fellow Health Protection Research Unit, Population Health Sciences, Bristol Medical School, University of Bristol, Bristol, UK; 4 Chair of department of psychiatry, Director of coordination of research centers, Mental Health Research Center, Iran University of Medical Sciences, Tehran, Iran

**Keywords:** Needs assessment, Sexual health, Sexual healthcare, Sexual education, Young adults, Iran, Tehran

## Abstract

**Background:**

Sexual Health and Relationships Education (SHRE) provides individuals with knowledge and skills to manage risky behaviors and take informed decisions to protect themselves against STIs, and unintended pregnancy. Only minimal SHRE is provided in Iranian schools and universities and previous research has highlighted needs and demands for improved SHRE and sexual services in Iran. This study explored young, Iranian adults’ experience of, and need for sexual health education, sexual skills training and sexual healthcare services, as well their views on how to augment and improve existing provision.

**Design and methods:**

Semi-structured interviews were conducted with a sample of 25 young adults who lived in Tehran, Iran and had volunteered to participate in the study. Transcripts were analyzed using thematic analysis.

**Results:**

Participants explained their needs and demands for sexual health education and sexual healthcare. They unanimously expressed their dissatisfaction with available SHRE and sexual health care provision. They highlighted barriers to gaining sexual health information and seeking advice and healthcare, including a lack of reliable resources, taboo and cultural barriers, lack of trust and protected confidentiality. This has resulted in ambiguities and misconceptions, including those regarding the cause and transmission of STIs and correct use of contraceptives. Participants recommended improvements, including holding mixed-gender extracurricular workshops with a comprehensive approach to sexual health and relationships education.

**Conclusions:**

There is a clear need and demand for provision of relevant and reliable sexual health and relationships education for young adults in Tehran. This should be addressed to empower young people to make informed choices and avoid risky sexual behavior.

**Supplementary Information:**

The online version contains supplementary material available at 10.1186/s12889-021-11371-x.

## Background

Tehran is the most populous city in Iran with a population of 8.5 million, including approximately 1.04 million people aged 18–25. Young people in Iran complete schooling at 18 and this is the legal age at which they can get married. Approximately 500,000 students are admitted into university courses in across the 10 universities in Tehran. So almost half of 18–25-year-old Tehranians attend university [12, 13]. There are, therefore, considerable opportunities to reach this age group with extra-curricular education and preventive services.

Sexual health and relationship education (SHRE) can provide knowledge, motivation and skills to help people to, (i) understand the potential consequences of their sexual behavior, (ii) make informed decisions about sexual relationships, (iii) more comfortably communicate about sex, sexuality and sexual health and (iv) protect themselves against sexually transmitted infections [1, 8].

SHRE is effective and does not promote earlier sexual debut. A review of reviews incorporating 37 systematic reviews (and 224 primary trials) indicated that comprehensive school based SHRE is effective in increasing knowledge, changing attitudes, and reducing risky sexual behavior [8]. Similarly, a review of 85 SHRE interventions for young people aged 15–24, delivered in schools, community centers and health clinics in the United States of America (USA) as well as developing countries, concluded that these interventions were effective and that there was no evidence indicating that SHRE is associated with earlier or more frequent sexual encounters [28]. Ideally, SHRE should promote “a state of physical, mental and social well-being in relation to sexuality… requiring a positive and respectful approach to sexuality and sexual relationships, as well as the possibility of having pleasurable and safe sexual experiences, free of coercion, discrimination and violence” (World Health Organization, n, d) [21].

SHRE has never been included in Iranian school curricula so sexual health knowledge is based on less regulated media and discussions with other young people [15]. Consequently, young adults can have poor sexual health knowledge [18, 23]. Many universities including those in Tehran, offer a single SHRE module (“Science of Family and Population”) which is a 20-h course taught by a lecturer specializing in religious and spiritual studies [14]. This module targets heterosexual people and does not include information relating to many elements of SHRE considered important by the World Health Organization (WHO) such as sexual consent, prevention of sexually transmitted infections, correct use of available contraception options and safer sexual practices across sexual orientations [29]. There are also pre-marriage classes teaching similar content and are compulsory for people getting married [19]. However, it is unclear how often such courses are run.

Free condoms are provided to sex workers and drug users in Tehran [17]. Other citizens can purchase contraceptives, including condoms, from pharmacies. However, because healthcare is predominantly privatized, such purchases are considered costly by middle class citizens and, especially by working class citizens, including young people. Thus, condoms can be purchased but the cost can be prohibitive [3]. There are a limited number of clinics across Tehran, referred to as “Centers for Behavioral Diseases”, that offer free and confidential testing for sexually transmitted infections (STIs) [2, 11]. However, these facilities are not publicly advertised and are used primarily by “high risk” groups such as drug users and sex workers, so attendance tends to be stigmatized.

A series of insightful studies have highlighted a need and demand, for, SHRE in Iran. For example, Shahhosseini and Hamzegardeshi [27] interviewed 77 young women, aged 11–19 and concluded that there is a strong demand for SHRE and that, in its absence from school curricula, young adults have turned to unreliable internet sources. Mosavi, Babazadeh, Mirzaii Najmabadi & Shariati [22] drew similar conclusions, based on interviews with adolescent girls and their mothers. These authors highlighted a lack of knowledge regarding sexual health, ease of access to unreliable and inaccurate information through the Internet, and evidence of increased risky, sexual behavior patterns among adolescents.

Mahmodi and Valiee [16] and Rahmati Najarkolaei, Niknami, Aminshokravi & Tavafian, [26] designed and delivered sexual health and STIs awareness programs aimed at Muslim women and university students, respectively. These studies recruited small and unrepresentative samples (60 married Muslim women and 109 female law or literature students at the University of Tehran, respectively) and used only pre-post-evaluations. The results are, nonetheless, encouraging because participants who completed these programs showed significant improvements in knowledge about HIV/STIs, although we do not know if this translated into changes to their sexual health protection.

Despite these encouraging findings, there is a lack of research into what exactly young Iranians know about sex and sexual health and what they want and need in terms of SHRE and sexual services. Moreover, research to date has not applied theoretically driven analyses to identify particular gaps in knowledge, motivation and skills which might be expected to shape population-level behavior patterns and could identify key SHRE targets. Such theoretical analysis could clarify exactly how improved services could impact perceptions, attitudes, motivations and behaviors relevant to improved sexual health among young people in Iran.

The Information-Motivation Behavioral skills model (IMB [9];), proposes that people need to be well informed, motivated and to have prerequisite skills to successfully change behavior patterns. The model was developed as a framework to improve interventions designed to promote HIV-preventive behavior and can be used to identify key targets for health promotion including, for example, accurate risk assessments, positive attitudes towards performing preventive actions, the perception that important others’ (e.g., partners)’ approve of protective actions and self-efficacy and skills relevant to protective actions. The model has been applied in the design and evaluation of effective HIV-preventive behavior [10].

### The present study

We aimed to clarify sexual health needs of young Tehranians by conducting needs-assessment interviews with young Tehranians, as recommended by the Intervention Mapping framework, [1, 4].

Applying the IMB, we sought to understand what these young people know, the cognitive bases of their motivation, what skills and training they might need and what services they valued and wanted. We defined four research aims.

1. To assess young people’s sexual health knowledge, and to identify their sources of sexual health information and available advice, as well as their recommended sources.
2. To explore young people’s beliefs, attitudes, norms and motivations in relation to sexual health protection.
3. To investigate the availability and accessibility of sexual health services for these young people.
4. To explore what additional sexual health services would be most valued by this group.

## Methods

Semi-structured, face-to-face interviews were conducted in person with 18–25-year-old Tehranians in a convenient location in Greater Tehran with one interviewer and one interviewee present. Participation was voluntary, and no incentives were provided to these key informants. Interviews took approximately 45 min and were conducted by the first author; a female doctoral candidate who has received training for interviewing young adults on sexual health. No relationship was established between the interviewer and the interviewees prior to interviews. The research was conducted in accordance with the Standards for Reporting Qualitative Research [24]. The study, and all data collection procedures, were approved by ethics committees of The University of Exeter Medical School and Iran University of Medical Sciences.

### Sampling and data collection

Convenience sampling involved placing advertisements in health clinics and university health centers in Tehran. Interested people were asked to contact the research team and were provided with a participant information sheet. Those who consented to participate were given details of the interview time and location by telephone. Interviews were conducted in quiet, private rooms in a hospital or university setting most convenient to the participant. Interviewees were made aware that they could withdraw at any time without giving a reason. Verbal consent (rather than written consent) was obtained from every participant to protect the interviewees’ anonymity. All participants consented to interviews being recorded and for quotations to be reported anonymously.

### Interviews

The semi-structured topic guide is provided as document 1 in Supplementary Materials. The guide included questions on (1) sexual health knowledge (2) perception of personal knowledge (3) content of any sexual health education (4) source of sexual and sexual health information (5) confidence in preventing STIs and protecting oneself in sexual relationships and (6) perceived accessibility of sexual healthcare and contraceptives. The topic guide was developed in accordance with the research questions and was pilot tested on 5 young adults. Interviews were conducted in Persian except for one, in which the interviewee requested use of English. Recorded interviews were transcribed verbatim, anonymized and translated, where necessary.

### Participants

One hundred and forty-five people responded to recruitment advertisements, of whom, 60 met the inclusion criteria of being Iranian and aged 18–25, speaking Persian as a first language and living in Greater Tehran. Thirty-five people declined to be interviewed when contacted or were not available during the data collection period. Twenty-five young women (*N* = 18) and men (*N* = 7) from various educational backgrounds, including those with high school diplomas (4), Bachelors degrees (13) and Masters degrees (8) were interviewed. Participants were from mixed socioeconomic backgrounds (high Income (9), middle Income (4) and lower Income (12). They identified as agnostic (11), atheist (1), theist (only believing in God) (10) and religious (identifying with a religion, including Islam and Christianity) (3). They were 18–25 years old, with the mean age of 23.

### Data analysis

NVivo was used to store transcripts and allocate excerpts to categories. A thematic analysis was conducted employing guidelines provided by Braun & Clark (2006) [7]. This involved 6 stages of analysis applied to anonymized interview transcripts. These included (1) establishing familiarity with the data (reading, re-reading and note taking) and (2) generating initial category definitions (sketching definitions and identifying content examples and recurring categories). Step 3 involved a more systematic search for themes and overlapping/corresponding content categories (checking if emerging definitions are applicable across interviews or are too general/specific). During step 4 the emergent categories were reviewed (checking that defined categories are distinct and correspond to multiple examples from interviews). Steps 3–5 involved multiple meetings between the first three authors to critically discuss each of the category definitions, the differences between categories and the appropriateness of each excerpt allocation to the category definition. The fifth step was to finalize the category definitions and the conceptual tree to which they belonged. Finally, interviews were re-read to ensure selection of all excerpts were relevant to the final category definitions and the results report written, was illustrative of the category tree, category definitions and the overall coding of transcripts [6].

To ensure validity, two researchers coded five interviews separately and reviewed themes. Many meetings were held in which the first three authors discussed transcript excerpts and identified appropriate thematic definitions. This resulted in collaborative interpretation and validation of textual categorization and facilitated detailed consideration of any differences of interpretation. The final themes and sub-themes were defined and reviewed by the first three researchers who discussed all selected quotes and their thematic allocations. The results of this coding are provided in document 2 of the Supplementary materials. The first author recorded reflective notes during data collection and revisited these through data analysis to ascertain inclusion and explanation of details expressed by participants.

## Results

Thematic analyses generated 12 themes, incorporating 32 sub-themes which are listed in document 2 in the Supplementary Materials. In total, 505 quotations were extracted from 25 interviews. Table 1 lists the main themes and numbers of quotations allocated to each theme. Approximately 80% of the interview transcript text was extracted as relevant quotations. All participants contributed multiple quotations across themes. Documents 3 and 4 provide the thematic map and all extracted quotations by theme and sub theme in the supplementary materials, respectively. These data show recurring content across interviews. It was notable that, in later interviews, little semantic refinement of the emerging thematic structure was added, suggesting that data saturation had been achieved. We concluded data collection after 25 interviews.

**Table 1:** 12 Main Themes

Theme	Number of Quotes
Sexual Health Knowledge and Perceptions of Personal Understanding	111
Used and Recommended Sources of Sexual Health Information	90
Availability and Quality / Content of Sexual Health Education	43
Understanding and Negotiation of Sexual Relationships	14
Concerns about Sexually Transmitted Infections (STIs)	22
Concerns about Pregnancy	21
Contraception and Condoms	65
Barriers to Using Sexual Health Services	66
Sexual Prohibition	19
Socioeconomic Sexual Health Inequalities in Tehran	13
Gender Power Inequalities in Sexual Relationships	12
Recommendations for Improved Sexual Health Education and Services in Tehran	29

Below we present a subset of illustrative quotations that highlight the core meaning of the 12 themes and highlight sub-thematic structure. We recorded demographic data including age, socioeconomic status and self-expressed religious beliefs, but we did not find differences in themes attributable to these individual characteristics.

### Theme 1 sexual health knowledge and perceptions of personal understanding

Most participants expressed a good knowledge of sexual organs and could name and describe male and female sexual organs, in contrast to their knowledge of STIs, symptoms and transmission methods. Although HIV and HPV were named, their symptoms and transmission methods were often unclear. Other STIs were sometimes named but again there was a lack of understanding of transmission routes (sub-theme 1i and 1ii).

Participants reflected on their level of sexual health knowledge compared to their peers. These assessments were categorized as similar to others (sub-theme 1iii), not as good as others (1iv) or superior to others (1v). While a few felt they knew more, most felt their level of knowledge was equal to their peers. Interviewees also noted that socioeconomic deprivation was likely to be associated with less sexual health knowledge (1vi).

### Theme 2. Used and recommended sources of sexual health information

The lack of official SHRE is likely to encourage self-education. Interviewees highlighted various sources of sexual health information and sources of sexual health information they would recommend to their friends. These overlapped considerably.

Six source categories were identified as sub-themes. The internet and social media (2i) were the most used and recommended sources of information. Interviewees mentioned popular social media apps like Instagram and Telegram along with Google as their source of sexual health information. By contrast, parents (2ii) were criticized for not discussing sexual health, although participants accepted that discussing sex was a taboo in their culture. Perhaps worryingly, pornography was identified as a learning tool by some male and female participants; with potential harms and informational benefits highlighted.

*Porn really did help me, because there wasn*’*t anything else that would show everything as real as it was. It helped me to see, understand and discover things. However, watching porn is not healthy as it might make you have unrealistic expectations from yourself or your partner.*

A few participants mentioned books (2iv) as a source of sexual health information, including general knowledge books (e.g., encyclopedias).

Some participants described their own sexual experiences as key learning opportunities (2v), and many trusted their friends’ descriptions and advice. Contrary to normative expectations, we found that some young women, but not men, mentioned experimenting with sexual relationships, in order to learn more about sex. Finally, although “doctors” were recommended as a useful source of information our interviewees did not report learning form this source (2vi).

### Theme 3. Availability and quality / content of sexual health education

Interviewees highlighted the very limited sexual health education provided in school, university and / or pre-marriage classes.

*We’ve never had an official sex education class.*

*I*’*ve learned everything I know through experience. No one has taught me anything.*

### Theme 4. Understanding and negotiation of sexual relationships

Interviewees discussed how young adults would manage sexual relationships. Quotes were categorized into four sub-themes (4i) Familiarity implies health in sexual partners, (4ii) Confidence and power in managing sexual relationships and (4iii) Communication in Sexual Relationships. These often highlight confidence in managing relationships despite lack of formal SHRE and developed self-management and behavioral skills.

*I usually try to talk about these kind of stuff before starting any relationship and would tell him about what I want before sex.*

*[Young people are] rarely … concerned about STIs because they trust their partners and believe that they haven*’*t been with unhealthy people.*

### Theme 5. Concerns about sexually transmitted infections (STIs)

Participants explained their concerns about STIs including (5i) Ambiguity and lack of education on STIs, (5ii) Invisibility of STIs, (5iii) Fear and worry about STIs and (5iv) Perception of other groups’ lack of concern. Some were not concerned because of their perceived skills while others expressed quite serious fears about STIs. Many interviewees acknowledged lack of reliable information and STIs invisibility as reasons for misconceptions and, at times, poor motivation to prevent STIs.

*We haven’t been educated for it and this can be as harmful as the diseases itself. We don’t consider STIs [to be] serious diseases.*

*The fact that you can hide your STI from others makes them not to be concerned about.*

### Theme 6. Concerns about pregnancy

Interviewees were more concerned about pregnancy than STIs, because pregnancy was visible and could lead to more serious life consequences. This was attributed to (6i) Fear and worry about pregnancy outside marriage and (6ii) Visibility of pregnancy leading to social and personal issues, especially because sex outside of marriage is not legal in Iran.

*I remember that it was around 1 or 2 years ago that we were in a gathering and one of my closest friends came by and she was like 100% sure that [she was] pregnant and we were all scared as hell, not because of the pregnancy itself but because of the consequences …*

### Theme 7. Contraception and condoms

Condoms were the most frequently mentioned contraception method with only a few participants identifying contraceptive pills as their preferred option. Interviewees discussed (7i) Condom availability and accessibility, (7ii) Condom cost, (7iii) Quality of Iranian condoms (7iv), Ease of condom use and (7v) Inconsistent use.

*[Condoms are] accessible everywhere. You can find them in both pharmacies and supermarkets. Therefore, the accessibility and availability are good.*

*In my opinion you*’*re better off not using condoms because you might be risking with a low quality one, maybe this way you would pull out because you don*’*t have that trust.*

*I think they are expensive. … For foreign condoms as imports are getting more complicated due to sanctions the prices are getting higher so they are more expensive.*

### Theme 8. Barriers to using sexual health services

Although there are government-funded sexual health centers in Tehran, our participants were not aware of them or how to access them. Participants also identified barriers in approaching doctors, including (8i) Cost of visiting doctors and sexual health care, and (8ii) Trust in doctors.

*I don’t know any sexual health clinics in Tehran … part of [the problem] is the lack of information on where to go, who to trust and spend … money on.*

*I have so many questions … and I can’t afford to visit a doctor to ask them.*

Interviewees reported reluctance to discuss their private lives, even when seeking advice or medical attention because they feared that these issues will be shared with their families or even law enforcement officials. Better relationships between young people and medical professionals might be facilitated by youth-friendly clinics in which confidentiality was explicitly guaranteed.

“*We are scared to tell the doctor about our issues, for example to tell them we’ve had sex out of marriage and they would let our families know about it. I*’*m absolutely terrified about that”*

Further personal and social barriers to seeking sexual healthcare in Tehran, include (8iv) Embarrassment as a barrier to sexual protection, (9v) Taboo shame and social disapproval as barriers, (9vi) Health motivation, and (9vii) Denial / fear.

*Some people are embarrassed to go and ask for condoms in a pharmacy because it*’*s usually out of hand reach and you should ask someone to give it to you. If it*’*s a lady selling it, it*’*s even worse for men, they would be even more embarrassed.*

### Theme 9. Sexual prohibition

Consistent with the identification of fear of social judgement as a barrier to seeking sexual health care, participants acknowledged that existing laws support social and cultural norms that portray sex as shameful or unacceptable for unmarried people and sanctify virginity in women.

*I know girls who give in to any form of sexual relationship other than the vaginal intercourse only to protect their virginity, it’s a huge concern for so many people to the extent they put themselves in painful positions to please the guy … to stay virgin.*

### Theme 10. Socioeconomic sexual health inequalities in Tehran

Interviewees highlighted socioeconomic inequalities in sexual health, highlighting that citizens from lower socioeconomic backgrounds face challenges in accessing and paying for sexual healthcare and contraception methods.

*And poor areas don’t have much of a choice, both with doctors, contraceptives and condoms.*

### Theme 11. Gender power inequalities in sexual relationships

Women interviewees indicated that they could not control heterosexual sexual encounters, including condom use, highlighting power inequalities and prioritization of male partners’ preferences; even when these young women were highly motivated to avoid STIs and pregnancy.

*There is this need to please guys in girls, and they tend to agree with whatever guys tell them, like not using condoms or having rough sex. I*’*ve seen this in my friends*’ *relationships.*

### Theme 12. Recommendations for improved sexual health education and services

All participants thought SHRE provision necessary and believed sexual health education would have optimal results if started from an early age. They suggested subjects such as contraception and condom use, sexual organs, pregnancy and relationship management skills to be included in short courses or workshops. Participants had varying opinions on the gender mix and delivery method of such programs.

*It should be started from the beginning of elementary school with teaching about sexual organs, then they should carry it on with sexual health in middle school.*

*In my opinion it would be better for the classes to be mixed gender, so that we all benefit from it equally.*

## Discussion

To our knowledge, this is the first qualitative assessment exploring sexual health education, training and service provision needs of 18–25-year-olds living in Tehran.

Participants expressed their demand for SHRE and shared recommendations for a potential intervention. Twelve themes and 32 sub-themes were identified from our thematic analyses of 25 interviews. These highlighted the demand for, and lack of SHRE, and provided in depth insight to existing sexual healthcare provision and needs. Interviewees also shared their understanding of SHRE and elaborated their unofficial sources of information. The interviews illustrated the negative consequences of poor SHRE, in a lack of understanding of sexual relationships, STIs and contraceptives and illustrated how inadequate knowledge influences sexual behavior.

We found that poor sexual health knowledge has caused misconceptions, and young adults have turned to unreliable sources, including their friends and social media, to find answers. This can deepen misconceptions and ambiguities regarding the threat, cause and treatment of STIs, the effectiveness of contraception methods, and the importance of consistent condom use. These findings correspond to those of Bostani Khalesi et al. [5].

Moreover, young adults deemed the quality and content of existing SHRE programs insufficient and ineffective and provided recommendations on ways to improve or augment current provision, which indicated their need and demand for comprehensive SHRE and mirrors the conclusions of Pourmarzi et al. [25].

Interviewees were unaware of existing government-funded sexual healthcare clinics and deemed visits to private doctors as limited by cost. They were concerned about confidentiality of their private information and the potential damage to their social image, even in conversations with doctors. Cultural barriers such as sexual prohibition and associated taboo and shame also discouraged visits to doctors.

Overall, the findings suggest a need for improved, well-advertised, accessible, confidential and reasonably priced sexual healthcare. Additionally, introduction of policies supporting patient confidentiality and pre-marriage sexual relationships would facilitate the removal of mistrust in current government-funded sexual healthcare services. Currently, contraceptives and condoms are not available for free, and long-acting reversible contraception (LARC) methods are only accessible through expensive, private clinics.

The information and skills foundation needed for effective STIs and pregnancy prevention was found to be underdeveloped in these young people. Interviewees acknowledged inconsistent condom use and, especially young women shared problems in negotiating protection. We concur with Mirzaee et al. [20] that such findings highlight a need for healthy, mutual relationship and sexual relations education and training for young Iranians. Our findings indicate that this should include, basic biology of STIs transmission, self-management skills (e.g., setting goals and priorities), relationship management skills and protection skills, including condom-acquisition, negotiation and use skills. Given the widespread use of internet sources, the creation of online training materials in Persian seems like an obvious first step to bridging this educational gap. This would also allow self-selection of short courses according to the needs of the users.

Young women expressed their lack of empowerment in managing sexual relationships, emphasizing the need for materials particularly addressing these issues. Our findings also suggest that presentations by young Iranians, similar to the target audience for such SHRE classes could optimize trust.

Our findings represent a novel needs assessment generating recommendations for improvement of health services and health education. Nonetheless, there are limitations to this research. We used a small sample and the applicability of our findings to other groups, including those who live in suburbs of Tehran, remains unclear. In addition, our sample may reflect selection bias because those who agreed to be interviewed may have been more confident, than average, in their sexual health knowledge and skills. Moreover, since the interviewees appeared to be predominantly heterosexual, our findings may not adequately reflect the needs, demands and experiences of individuals with other sexual orientations and preferences.

A large-scale quantitative study with quotas set for respondent types could generate a more representative portrayal, including participants from diverse socioeconomic and sexual backgrounds and those with special requirements (e.g., those with physical disabilities and learning difficulties and physical, limiting conditions). Such large-scale work could underpin tailored versions of SHRE and service provision for identified sub-groups. We found no discernible patterns, indicating differences in views across educational, socioeconomic and religious backgrounds, perhaps due to the almost universal reliance on internet and social media sources.

Notwithstanding these limitations, our study presents insights and recommendations for the development of sexual health services and education for young people in Tehran and provides a good basis for developing and testing preliminary materials, incorporating learning from international developments. Additionally, our findings could provide guidance to policy makers about service and educational gaps that could prompt revision of SHRE and sexual healthcare provision for young Iranians, as recommended above. It would be interesting to explore young people’s reflections and recommendations in relation to service provision with policy makers and health care practitioners.

## C**onclusions**

In conclusion, sexual health knowledge is poor amongst young Tehranians and they do not perceive sexual healthcare as available and accessible. Young adults want comprehensive SHRE to understand and manage sexual health risks and conduct their sexual relationships safely. They requested non-judgmental, confidential, accessible, and reasonably priced sexual healthcare. This study (including findings reported in the supplementary document) highlights the problems young Tehranians face daily in managing their sexual health. These findings provide a blueprint for sexual health educational and service enhancement that would meet these needs.

## Supplementary Information

**Additional file 1.**

## Data Availability

Data available in article supplementary material: The detailed data that supports the findings of the thematic analyses are available in the supplementary material of this article.

## Abbreviations

SHRE: Sexual health and relationship education
UNESCO: United Nations Educational, Scientific and Cultural Organization
USA: The United States of America
WHO: The World Health Organization
