# Supplementary material for: A qualitative assessment of the sexual-health education, training and service needs of young adults in Tehran
Source: BMC Public Health. 2021 Jul 13;21:1386. doi: 10.1186/s12889-021-11371-x (PMC8276474; doi:10.1186/s12889-021-11371-x)
Supplement: Supplementary file 1 — Additional file 1. [file 12889_2021_11371_MOESM1_ESM.docx]

Supplementary Documents: “A qualitative assessment of the sexual health education, training and service provision needs of young adults in Tehran”

Contents Page

Supplementary Document 1: Interview Protocol 2

Supplementary Document 2: 12 Main Themes 6

Supplementary Document 3: Thematic Map 7

Supplementary Document 4: Quotes grouped by themes and sub-themes 13

Supplementary Document 1: Interview Protocol

Interview Topic Guide for

“A qualitative assessment of the sexual health education, training and service provision needs of young adults in Tehran**”**

Asked to confirm consent before each interview:

Can you confirm that you have read the participant information sheet I have provided and that you are aware that you can stop the interview at any time without explaining why.

Can you confirm that you understand that I do not wish to know your name and all data will

be anonymized and when we combine data across interviews in our analyses?

Can I confirm that you are happy for me to record this interview?

Before we begin, I want to clarify that, while I am interested in your view of sexual health education and sexual health services and how useful these are to you, I am not going to ask you any questions about your sexual preferences. My research is not concerned with individual’s sexual behavior. I am interested in education and services in Tehran that are relevant to sexual health. So I will be asking you about how you have experienced such education and services.

I am going to ask you questions about sexual health education and training, including education and training that you have received and any education or training you feel would be beneficial to you. Do you also understand that I will audio record the interview and that you can refuse to answer any question and end the interview at any time?

1. Can I ask you a few questions about your background, but not your name or anything specific that could identify you?
2. Which age group are you in?
3. How long have you lived in Tehran?
4. Which district do you live?
5. What is your educational background?
6. How would you describe your spiritual and / or religious beliefs?

3. How well educated do you think young adults are in Tehran in relation to their sexual health?

4. How concerned do you think young adults are in Tehran are about unwanted pregnancy and/or sexually transmitted infections?

5. What sexual health education have you received and who provided this?

1. Can you tell me about the topics that were included in this sexual health education?
2. How useful and comprehensive would you say this sex education was?
3. Are there any particular topics that you would have liked to have more coverage of?

6. Do you think you know more or less than a typical 18–25-year-old in Tehran about sexual health risks and how to maintain sexual health?

7. Where did you obtain the majority of your knowledge about sexual health (e.g., school, peers, etc.)?

8. How confident do you feel that you can prevent unwanted pregnancy or STI in your present or future sexual relationships?

9. Do you think sexually active young adults are in Tehran are protecting themselves against sexually transmitted infections?

1. If so, how?

10. Do you think sexual health education in Tehran needs to be extended or changed?

1. If so, how?

11. At what age do you think sexual health education should begin?

1. Should some topics be delivered at later developmental stages? What would you suggest?

12. How familiar would you say a typical 18-25 Tehranian is with the anatomy and function of their own sexual organs and those of the opposite sex?

1. Would you be able to name male and female sexual organs?

13. How confident and in control do you think a typical 18–25-year-old Tehranian is in managing sexual relationships?

14. Thinking about sexually transmitted disease, would you be able to provide me with examples of common sexually transmitted infections that a young adult would want to protect themselves from?

a. How is each infection transmitted? How many people do you think are affected by each infection/ disease in Iran?

15. What contraception methods do you or your partner currently use or plan to use in your future relationships? Why?

16. How accessible do you find contraceptive methods and sexual healthcare for young adults in Tehran?

17. Can you think of any reasons that young adults would avoid seeking sexual health knowledge and/or sexual healthcare in Tehran?

18. If you were advising a friend on how to improve their sexual health knowledge what would you recommend?

19. If you were advising a friend worried about having a sexually transmitted infection or an unwanted pregnancy what would you recommend?

20. Do you regard sexual health care (including contraception pills, condoms, educational materials, visits to doctors etc.) inexpensive or expensive? Do you think the cost is justified?

21. What would you top five recommendations for improved sexual health education and training in Tehran?

22. Would you like to add anything more?

23. Thank you very much for your time. How did you find participating in our study? Do you have any further questions?

Supplementary Document 2: 12 Main Themes

1. Sexual Health Knowledge and Perceptions of Personal Understanding
2. Used and Recommended Sources of Sexual Health Information
3. Availability and Quality / Content of Sexual Health Education

4. Understanding and Negotiation of Sexual Relationships

5. Concerns about Sexually Transmitted Infections (STIs)

6. Concerns about Pregnancy

7. Contraception and Condoms

8. Barriers to Using Sexual Health Services

9. Sexual Prohibition

10. Socioeconomic Sexual Health Inequalities in Tehran

11. Gender Power Inequalities in Sexual Relationships

12. Recommendations for Improved Sexual Health Education and Services in Tehran

Supplementary Document 3: Thematic Map

Below, the main themes identified from the analyses are described and all sub-themes are listed. There were 12 themes and 32 sub-themes.

**1. Sexual Health Knowledge and Perceptions of Personal Understanding** (six sub-themes)

*In general, sexual health knowledge was insufficient amongst all participants. This is reflected in quotes regarding knowledge of STIs and sexual organs and their misunderstandings about different aspects of sexual health and relationships. Participants also reflected on their level of knowledge compared to their peers.* *Quotes were categorized into six sub-themes.*

1. Knowledge of STIs; symptoms and transmission
2. Knowledge of sexual organs
3. Similar to others
4. Inferior to others
5. Superior to others
6. Deprivation and knowledge of sexual health

**2. Used and Recommended Sources of Sexual Health Information** (six sub-themes)

*Interviewees highlighted various resources of sexual health information. While Internet and social media were the most quoted, other sources of information were also mentioned. Quotes were categorized into six sub-themes.*

- 1. Internet and social media
  2. Parents
  3. Pornography
  4. Books
  5. Personal or friend’s experience
  6. Doctors

**3. Availability and Quality / Content of Sexual Health Education**

*Interviewees expressed various opinions regarding sexual health education which was provided at school, university or pre-marriage classes.*

**4. Understanding and Negotiation of Sexual Relationships** (three sub-themes)

*Interviewees expressed understandings of sexual relationships and how to negotiate their needs. In these quotes, interviewees have commented on matters such as their level of confidence and power in managing sexual relationships. Quotes were categorized into three sub-themes.*

1. Familiarity implies health in sexual partners
2. Confidence and power in managing sexual relationships
3. Communication in Sexual Relationships

**5. Concerns about Sexually Transmitted Infections (STIs)** (four sub-themes)

*This theme illustrates interviewees concerns with STIs. Some concerns were caused by lack of sexual health education, e.g., ambiguity and lack of education on STIs, while others were created as a result of personal choices and social behaviors, e.g., multiple Partners and STIs. Quotes were categorized into four sub-themes.*

1. Ambiguity and lack of education on STIs
2. Invisibility of STIs
3. Fear and worry about STIs
4. Perception of other groups’ lack of concern

**6. Concerns about Pregnancy** (two sub-themes)

*Interviewees were generally more concerned with pregnancy than STIs. These concerns were recorded in quotes categorized into two sub-themes.*

1. Fear and worry about pregnancy outside marriage
2. Visibility of pregnancy leading to social and personal issues

**7. Contraception and Condoms (**five sub-themes)
 *Interviewees expressed their views about STIs and pregnancy prevention methods' availability, cost and quality. They identified condoms as the most popular contraceptive method. Quotes were categorized into five sub-themes.*

1. Condom availability and accessibility
2. Condom cost
3. Quality of Iranian condoms
4. Ease of use
5. Inconsistent use

**8. Barriers to Using Sexual Health Services** (six sub-themes)

*Interviewees expressed their views on the availability of sexual health services, including sexual health clinics.* *They mentioned doctors as a source of sexual health knowledge but doctors were also deemed as not trustworthy by some participants. Interviewees also identified various psychological barriers to seeking sexual healthcare in Tehran, including personal and social barriers. Quotes were categorized into six sub-themes.*

1. Cost of visiting doctors and sexual health care
2. Trust in doctors
3. Embarrassment as a barrier to sexual protection
4. Taboo shame and social disapproval as barriers
5. Health motivation
6. Denial / fear

**9. Sexual Prohibition**

*Interviewees commented on social and cultural norms and behaviors which portray sex as shameful or unacceptable for unmarried people.*

**10. Socioeconomic Sexual Health Inequalities in Tehran**

*Interviewees highlighted inequalities in sexual health and service availability and accessibility in Tehran based on socioeconomic status.*

**11. Gender power inequalities in sexual relationships**

*Interviewees commented on women’s empowerment, or lack of it in sexual relationships.*

**12. Recommendations for Improved Sexual Health Education and Services in Tehran***Interviewees offered various recommendations on how sexual health education and services in Tehran could potentially be improved.*

Supplementary Document 4: Quotes grouped by themes and sub-themes

Below all quotes extracted from the interviews are categorized into the identified themes and sub-themes. Overall, these quotes represented 80% of the text in transcribed interviews. The number of quotes in each of the 12 themes was as follows : Sexual Health Knowledge and Perceptions of Personal Understanding [111], Used and Recommended Sources of Sexual Health Information [90], Availability and Quality / Content of Sexual Health Education [43], Understanding and Negotiation of Sexual Relationships [14], Concerns about Sexually Transmitted Infections (STIs) [22], Concerns about Pregnancy [21], Contraception and Condoms [65], Barriers to Using Sexual Health Services [66], Sexual Prohibition [19], Socioeconomic Sexual Health Inequalities in Tehran [13], Gender power inequalities in sexual relationships [12] and Recommendations for Improved Sexual Health Education and Services in Tehran [29].

The following annotation is used: Participant [P], Female [F], Male [M], Agnostic [AG], Atheist [AT], Theist [TH], Religious [R], High Income (HI), Middle Income (MI) and Low Income (LI).

1. **Sexual Health Knowledge and Perceptions of Personal Understanding**

Mainly in response to questions 6,12 and 14 from the interview protocol.

14. Thinking about sexually transmitted disease, would you be able to provide me with examples of common sexually transmitted infections that a young adult would want to protect themselves from?

1. How is each infection transmitted? How many people do you think are affected by each infection/disease in Iran?

12. How familiar would you say a typical 18-25 Tehranian is with the anatomy and function of their own sexual organs and those of the opposite sex?

1. Would you be able to name male and female sexual organs?

6. Do you think you know more or less than a typical 18–25-year-old in Tehran about sexual health risks and how to maintain sexual health?

1i: Knowledge of STIs; symptoms and transmission

*HIV,HPV,Syphilis. (p8,F,25,AG,* *HI)*

*Gonorrhoea, Syphilis. (p7,M,21,TH,* *HI)*

*HPV, Gonorrhea, AIDS. (p5, F, 25, AG, MI)*

*AIDS, Hepatitis C, Gonorrhoea. (p4, M, 25, TH, LI)*

*Gonorrhea, HPV and Aids. (p3, F, 20, AG, HI)*

*Gonorrhea. Syphilis. HIV. (p25,F,25,R,* *LI)*

*HIV can be transmitted through using shared needle and also sex. I think during sex when the guy ejaculates, the transfer of such fluid into the female's body will transfer the HIV virus. (p24, F, 22, AG, LI)*

*AIDS, HPV which I’ve heard is becoming really common these days especially among Iranians. (p24, F, 22, AG, LI)*

*AIDS, HPV. (p23, F, 20, AG, LI)*

*Gonorrhea, AIDS, Hepatitis. (p21, M, 25, AG, HI)*

*AIDS, HPV. (p20, F, 23, AT, HI)*

*AIDS. (p2,F,18,AG,* *HI)*

*HPV is being talked about a lot recently. I also know about HIV. (p19, F, 24, TH, HI)*

*HIV, HPV and Hepatitis. (p18, F, 18, AG, HI)*

*Gonorrhea and AIDS. (p17, M, 19, TH, HI)*

*AIDS, Hepatitis, HPV, herpes. AIDS gets transmitted through blood and sex. Don't know about the others. (p16, F, 25, AG, LI)*

*AIDS, Hepatitis, Gonorrhea. (p14, F, 25, R, LI)*

*AIDS, Hepatitis, HPV and Gonorrhea. (p15, F, 25, TH, LI)*

*Hepatitis, AIDS, HPV. (p13, F, 25, TH, LI)*

*HIV can get transmitted through blood for example through oral sex, if there is an open wound in my mouth the virus can get transmitted to my body. It’s not just sex it can also get transmitted through using contaminated needles and blades. (p12, F, 25, TH, MI)*

*HIV, HPV. (p12, F, 25, TH, MI)*

*HPV, Syphilis. (p11,F,25,TH,* *LI)*

*I only know HIV. (p10, M, 24, TH, LI)*

*HIV. (p10, M, 24, TH, LI)*

*AIDS. (p1, F, 22, R, MI)*

*HPV, delayed ejaculation, decreased sex drive, orgasm related issues, and skin related diseases, Fungi, Varicocele. (p6, M, 25, AG, MI)*

*Gonorrhea, Syphilis, that’s all. I don’t know how they get transmitted; I think from anal sex. Fortunately, I don’t prefer anal sex so I’m not going to get these diseases. (p15, F, 25, TH, LI)*

*For gonorrhea I think it gets transmitted through intercourse. I’ve read about gonorrhea on the internet, Men can get it, but it doesn’t get transmitted to women. I don’t know exactly how though. (p14, F, 25, R, LI)*

*Gonorrhea I think is transmitted orally and from mother to child. (p15, F, 25, TH, LI)*

*I believe UTI mostly comes from having sex. (p21, M, 25, AG, HI)*

*Although HPV is related to cleanliness in my opinion. (p24, F, 22, AG, LI)*

*I believe women who are HIV positive in Iran are way more than men. Maybe around 40% of women who are in bad conditions in Iran are HIV positive. By bad conditions I mean women who are careless with their sexual relationships or who are addicted because these are the ones who would get into any kind of sexual activity. (p24, F, 22, AG, LI)*

*I believe HIV rates are lower among men. (p24, F, 22, AG, LI)*

*Addicted people usually get both Hepatitis and AIDS. I’ve heard in foreign countries a cure has been found for it. We don’t have it here in Iran, I guess. If you get AIDS in Iran, there’s no cure or treatment for you. (p21, M, 25, AG, HI)*

*AIDs which the chances of getting it is way more unlikely than the other STIs. Chances of getting AIDS is really low, even if your partner has AIDS, chances of you getting it is really low. (p21, M, 25, AG, HI)*

*I don’t know if it was syphilis or Lupus. (p1, F, 22, R, MI)*

1ii: Knowledge of sexual organs

*I have balls, 2 of them, I have a penis then my prostates which is behind all of these. They have a vagina, I don’t know their names, they have this thing that they urinate from, ovaries and uterus but I don’t know where they are placed. (p10, M, 24, TH, LI)*

*Vagina, Clitoris, Anus. Balls. (p11,F,25,TH,* *LI)*

*Vagina, Uterus, Ovaries and Clitoris, Testicles and Penis. (p12, F, 25, TH, MI)*

*Vagina, Penis, Anus. (p13, F, 25, TH, LI)*

*Vagina, Ovaries, Uterus. Penis, Testicles. (p14, F, 25, R, LI)*

*Uterus, Ovaries. Anus, Testicles, Prostate. (p15, F, 25, TH, LI)*

*Uterus, Ovaries. Testicles, Prostate, Penis. (p16, F, 25, AG, LI)*

*Penis, Anus.Vagina. (p17,M,19,TH,* *HI)*

*Clitoris, Vagina, Anus, Uterus and ovaries. Penis and Testicles, Prostate. (p18, F, 18, AG, HI)*

*Vagina, Ovaries. 2 Testicles and Penis. (p19, F, 24, TH, HI)*

*Clitoris, Urethra, Vagina, Anus, the G-spot is in the uterus. 2 testicles, penis, prostate is inside testicles. Male’s anus is their G-spot, inside their anus. (p2, F, 18, AG, HI)*

*Vagina, Uterus, Ovaries. Testicles, Penis, Prostate which is something that is placed near the anus. (p20, F, 23, AT, HI)*

*Prostate, Testicles, Penis, Clitoris, Uterus. Women’s prostate is their G-spot, but I don’t know where it located is. (p21, M, 25, AG, HI)*

*Vagina, Clitoris. Testicles, Penis. (p21,M,25,AG,* *HI)*

*Vagina, Ovaries, Uterus. Testicles, Prostate which is inside the testicles. (p23, F, 20, AG, LI)*

*Vagina, Hymen, Ovaries. Testicles, Penis, Prostate which is obviously inside or maybe it is near the Testicles. (p24, F, 22, AG, LI)*

*Ovaries, Uterus, Urethra, Vagina, Anus. Prostate, Penis and Testicles. (p25, F, 25, R, LI)*

*Penis, Testicles, Uterus, Ovaries, Anus, Vagina, Urethra. (p4, M, 25, TH, LI)*

*I don’t know, there are 2 holes, one is for sex and the other one is for urinating, Umm, That’s it! And clitoris. They have Testicles, 2 of them and a hole for urinating and ejaculation and that’s all. I don’t know where the prostate is located but I’ve heard of it. (p5, F, 25, AG, MI)*

T*he penis. Anus, Urinary tract, Vagina. (p9, M, 22, TH, LI)*

*Uterus, Ovaries, Clitoris, Labia and Urethra. Vagina leads to the uterus and has the g-spot in it. Penis, testicles which produce sperms, their prostate which is placed between their testicles. (p3, F, 20, AG, HI)*

*Penis, 2 testicles, prostate which is close to the testicles. Clitoris, vagina which they also urinate from, anus. (p6, M, 25, AG, MI)*

*Penis, 2 testicles. I don’t know where the prostate is, I think it must be somewhere around testicles. Vagina, Ovaries are above the uterus, urinary tract, clitoris and labia. (p7, M, 21, TH, HI)*

*Ovaries, Uterus, Clitoris, G spot, Vagina, Hymen, Anus and Urethra. Penis, Testicles, Prostate which is behind the testicles. (p8, F, 25, AG, HI)*

*I think our level of knowledge is the same, but some guys don’t know that women’s urethra is separated from their vagina, they think we urinate from our vaginas. Because they have only one way out on their penis, they think we are the same. (p8, F, 25, AG, HI)*

*Prostate which is a vein under the testicles. (p9, M, 22, TH, LI)*

*Anus, Uterus. We get to the uterus through the anus, and we urinate from the vagina. The prostate which is the hole (she then drew a picture, and it was understood that she thought of anus as prostate.). Women also have prostate, but I’m not sure where it is. (p1, F, 22, R, MI)*

*Penis, Anus, the Prostate gland hangs among the balls at their front. (p13, F, 25, TH, LI)*

*G Spot and Ovaries. (p19, F, 24, TH, HI)*

*Clitoris, Urethra, Vagina, Anus, the G-spot is in the uterus. (p22, F, 25, AG, LI)*

… *Prostate which is placed in men’s thighs. (p14, F, 25, R, LI)*

*Vagina, Hymen, Ovaries. (p24, F, 22, AG, LI)*

*Testicles, Penis, Prostates which is obviously inside or maybe it is near the Testicles. (p24, F, 22, AG, LI)*

*Testicles, Prostate which is inside the testicles. (p19, F, 24, TH, HI)*

*Clitoris, Uterus. Woman’s prostate is their G-spot, but I don't know where it located is. (p21, M, 25, AG, HI)*

1iii: Similar to others

*I think our level of knowledge is the same. (p8, F, 25, AG, HI)*

*I guess we all know about the same. (p5, F, 25, AG, MI)*

*I think we all have the same level of knowledge. (p12, F, 25, TH, MI)*

*I don’t think some know more than the others, everyone’s knowledge is about the same level because no one has ever been educated for this. (p4, M, 25, TH, LI)*

*I think we all know the same. (p12, F, 25, TH, MI)*

*We probably all know the same. (p24, F, 22, AG, LI)*

*[On knowledge of sexual organs] I guess we know all the same. (p21, M, 25, AG, HI)*

*I know more about the anatomy of men and girls know more about their own bodies. It also depends on if the person has done some research or not. Sexual orientation matters too. Like I’m gay and don't really care what happens in women’s bodies. (p17,M,19,TH,* *HI)*

*[On knowledge of sexual organs] We all know the same. (p1, F, 22, R, MI)*

*I think we all know the same. (p15, F, 25, TH, LI)*

*I believe we all know the same. (p16, F, 25, AG, LI)*

*I think we all know the same. (p14, 25, F, R, LI)*

*Amongst normal people, we all know the same. (p12, F, 25, TH, MI)*

1iv: Inferior to others

*Others know much more definitely. I don’t know that much. But no-one is 100% familiar with the opposite sex’s body. (p5, F, 25, AG, MI)*

*Others know better than I do. (p24, F, 22, AG, LI)*

*Friends and people around my age know more. Because they are in relationships and such things become issues for them, so they have done more research about it, that’s why they have more information. (p23, F, 20, AG, LI)*

*Because I don’t know much, I guess others know better. (p22, F, 25, AG, LI)*

*It depends, If the person is single, they might not notice or care about these things however if they are in a relationship, they might even know more than me. (p20, F, 23, AT, HI)*

*I think my friends know more than me because I don't know much. (p19, F, 24, TH, HI)*

*I have a friend that hasn’t even kissed a boy at this age but knows more than me. So, I don’t think it depends on if you do it or not. I think my friends have more information than I do. (p19, F, 24, TH, HI)*

*I’m somewhere in between, some might know more than I do. (p18, F, 18, AG, HI)*

*Others know better than I do. (p15, F, 25, TH, LI)*

*[On knowledge of sexual organs] Others probably know better. (p13, F, 25, TH, LI)*

*I know less than others. (p1, F, 22, R, MI)*

*I don’t know much about sexual organs; others must know much better. (p10, M, 24, TH, LI)*

1v: Superior to others

*Those who study in fields of medical sciences or biology, they definitely have more information than I do. (p12, F, 25, TH, MI)*

*I think I know more than others. Others know less. (p7, M, 21, TH, HI)*

*I believe I know relatively more, taking the full range of knowledge at 100%, I know 50% and other young adults know 30%. (p3, F, 20, AG, HI)*

*I think I know more than my friends. There might be some people in my friends’ circle that have much more knowledge than me but overall, I can claim I know much more. (p25, F, 25, R, LI)*

*I know more than others, same as my partner. I know more about stuff that are related to men and my partner knows more about women. Compared to others I know much more however my friends know about some stuff by experience. Experience equals knowledge. (p21, M, 25, AG, HI)*

*I almost know 80% of my own body. But not everyone knows as much. (p2, F, 18, AG, HI)*

*Compared to people around me I know more. (p13, F, 25, TH, LI)*

*I’m sure I know about 40% of it. I can’t claim I know more than other young adults, amongst people whom I hang out with, I know more than the others. Mostly I know more than the others. (p2, F, 18, AG, HI)*

*I guess I am good where I am, but I can also see other people who have less information. (p11, F, 25, TH, LI)*

*I would say the educated people or umm I don’t know like those whom their parents are in educational system or are doctors, those are the minority that know about this stuff. (p10, M, 24, TH, LI)*

1vi: Deprivation and knowledge of sexual health

*The main problem for people in deprived areas who don’t use protection is lack of knowledge. If they knew it’s good for them, they would have bought it. For example, when I tell my friend to be careful, she already has a baby at the age of 22, she would say never mind if god wants to give you a child he would, it is out of our hands. (p24, F, 22, AG, LI)*

*The district you live in plays a determinant role, people in deprived areas are more likely to have unprotected sex that’s why the stats for unintended pregnancy is much higher in these areas. Poverty is a big factor; it causes limitation of options and education. (p25, F, 25, R, LI)*

*Some people from the deprived parts of the society might question why they should pay for such things at all. When prioritizing their issues and costs, they don’t see any reason to pay for such things. Cultural and educational background plays an important role. (p9, M, 22, TH, LI)*

*In deprived areas the culture is a bit different, and they are more conservative. (p19, F, 24, TH, HI)*

*In the affluent districts people easily go and ask for condoms because they have accepted the fact that they need to use them for sake of their own health and protection but the belief that bad things only happen to the others is growing among poorer people. (p5, F, 25, AG, MI)*

**2.** **Used and Recommended Sources of Sexual Health Information**

Mainly in response to question 7,18 and 19 from the interview protocol.

7. Where did you obtain the majority of your knowledge about sexual health (e.g., school, peers etc.)?

18. If you were advising a friend on how to improve their sexual health knowledge what would you recommend?

19. If you were advising a friend worried about having a sexually transmitted infection or an unwanted pregnancy what would you recommend?

2i: Internet and social media

*Searching on the internet. For example, I used to search sex and how to do it, the key words I used were sex or women’s climax and orgasm, or how to make your partner satisfied in sex so that I can be a better partner in sex. I tried to search and learn more about women because it is not only about reaching orgasm yourself, making your partner satisfied is more important because men can reach orgasm much easier, but it can happen very often that women don’t reach orgasm. Also, I wanted to last longer. Things like that. So, internet was my one and only source and answered many of my questions. (p10, M, 24, TH, LI)*

*I was researching as I was studying English, I used to search in English, so my sources were online, and I searched about STIs, the pregnancy process, what do guys like what do girls like. (p11, F, 25, TH, LI)*

*I searched about cons of having anal sex and read a lot about it or chances of getting pregnant or delayed periods. (p12, F, 25, TH, MI)*

*I didn’t receive any education, I searched about it, but it was after my first sexual experience. I read things online and gained the information I needed. (p15, F, 25, TH, LI)*

*I don’t know of any reliable sources. I got all my knowledge from the internet. (p8, F, 25, AG, HI)*

*Every time I have an issue, I go and search about that specific subject on the internet. (p22, F, 25, AG, LI)*

*I read a lot about STIs on the internet however I haven't read any books about them. This kind of books can be found but I have never tried to find and read them. (p22, F, 25, AG, LI)*

*We haven’t received any education however we have learned some stuff on our own. By googling, if something becomes a question, we would google it. (p22, F, 25, AG, LI)*

*I get most of my information from social media. I haven't read any book about this subject. By social media mostly I mean Telegram and Instagram. In Instagram I follow pages about women’s health. (p23, F, 20, AG, LI)*

*I’ve googled the things I know. (p18, F, 18, AG, HI)*

*I got the more accurate information from the internet. (p20, F, 23, AT, HI)*

*Stuff that I googled about were things like the natural cycle. This is the last thing I searched on the internet. I haven’t read books or watched videos. (p25, F, 25, R, LI)*

*In my opinion if you know how to search on the internet properly and you speak good English, you’re good, as you can find reliable sources in this field. That would be great. If you read articles from random websites in Persian it wouldn’t be really helpful and reliable. (p5, F, 25, AG, MI)*

*It was on Instagram from pages like “Organic minded”. I’ve found it recently, the content is great, and I read it and take screenshots and send them to my partner so he would read them too. (p20, F, 23, AT, HI)*

*I mostly searched on the internet. For instance, in my Telegram, I have this channel called “Scientific Sexology Team” Some of the stuff this channel shares are really helpful, so I share it with my friends. I used to google my questions when I was younger but now, I don’t need to. I don’t know, maybe someday I need it again. I used to search these: How do you get pregnant? How can you prevent it? How can you show your love? How can you satisfy your partner? (p24, F, 22, AG, LI)*

*After the age of 20 when the person gets in a relationship, they would start searching about it on the internet, YouTube or websites like that, they gain this information on their own, it’s not like there’s a class for it. (p21, M, 25, AG, HI)*

*I’ve found an Instagram page which belongs to a lady who is a psychiatrist and talks about all these sexual health and relationship stuff. That helps a lot. (p3, F, 20, AG, HI)*

*Those with a bit of more knowledge have learned through googling or other forms of searching resources. They’ve done it all by themselves, not so much from the family or school. (p5, F, 25, AG, MI)*

*I searched the internet. (p16, F, 25, AG, LI)*

*I’ve got the information I have now from books and the internet. As far as I know it’s the same with my friends. (p8, F, 25, AG, HI)*

*What we know is either from experience or searching on the internet. We have earned all the knowledge we have by ourselves. (p22, F, 25, AG, LI)*

*There wasn’t any sexual health education in school, anything I know now I’ve learnt from the internet or my friends. It all depends on how much you have researched on your own. (p22, F, 25, AG, LI)*

*I would tell them to stick to the internet. Nothing else. (p10, M, 24, TH, LI)*

*Search online and read about it. (p12, F, 25, TH, MI)*

*There’s this page in Instagram which I follow: Organic minded. I would introduce that to her. (p11, F, 25, TH, LI)*

*Internet search. (p15, F, 25, TH, LI)*

*I would tell them to search the internet. (p17, M, 19, TH, HI)*

*Search the internet. (p24, F, 22, AG, LI)*

*Search the internet. (p18, F, 18, AG, HI)*

*Search the internet. (p16, F, 25, AG, LI)*

*It depends on the problem they have. I would ask what their problem is. I don’t know about everything, but I would suggest him to search the internet. (p17, M, 19, TH, HI)*

*I would recommend them to search the internet. Also, I would tell them to take care of their health. (p19, F, 24, TH, HI)*

*If I don’t know any solutions, I would search and help her. (p19, F, 24, TH, HI)*

*I believe the internet is the only way that can help us in Iran. (p20, F, 23, AT, HI)*

*I would send them stuff on Telegram for them to see. I mean I would tell them to search online. I would have asked them how much they know and then based on their answer I would have sent them what I have found useful on WhatsApp or Telegram. When they become eager enough, they will go and search further for themselves. (p21, M, 25, AG, HI)*

*I would tell him/her to search it on the internet. (p9, M, 22, TH, LI)*

*I don’t know of any reliable sources. I got all my knowledge from the internet; I don’t know of any legit source to recommend. (p8, F, 25, AG, HI)*

*Search their issues on the Internet. (p6, M, 25, AG, MI)*

2ii: Parents

*Parents also don’t discuss it. Neither parents nor schools would teach us anything. (p20, F, 23, AT, HI)*

*Parents also haven’t discussed it with us. (p23, F, 20, AG, LI)*

*Parents don’t inform their kids about this subject, so you start to notice and find out things from talking to your friends. (p4, M, 25, TH, LI)*

*They said you would find out yourself. Mostly the adults, including teachers and parents wouldn’t welcome such topics. (p1, F, 22, R, MI)*

*Parents wouldn’t talk about it. (p10, M, 24, TH, LI)*

*I didn’t learn anything from my parents or a person who would know professionally. Because it’s a taboo to speak of and it sounds really bad and ugly to our parents. (p7, M, 21, TH, HI)*

*Parents also don’t talk about it. There aren’t so many people whom their parents would teach them or talk to them about this stuff. (p8, F, 25, AG, HI)*

*I feel people my age haven’t learned anything from their parents. (p9, M, 22, TH, LI)*

*Again, our parents have not taught us much. (p9, M, 22, TH, LI)*

*My parents never talked to me about it. They are not conservatives however they never brought up the conversation. (p21, M, 25, AG, HI)*

*This should start within families but unfortunately it is a taboo to speak of within the family members, so they don’t teach their kids. (p10, M, 24, TH, LI)*

2iii: Pornography

*I haven’t read a book about it I have only watched porn. (p6, M, 25, AG, MI)*

*Before I started dating, I used to learn from watching porn. (p7, M, 21, TH, HI)*

*I watched porn. Porn really did help me, because there wasn’t anything else that would show everything as real as it was. It helped me to see, understand and discover things. However, watching porn is not healthy as it might make you have unrealistic expectations from yourself or your partner. (p5, F, 25, AG, MI)*

*I learned about different positions and how we have sex from porn. In my opinion porn can be educative. (p17, M, 19, TH, HI)*

*I started hating my parents for a while when I learned that I am a result of such relationship, and it was so weird to me to find out my parents have done things to each other that you see in porn movies. Before that I had watched some porn and I used to think it is a bad thing to do. Then I learned that creation is based on such things. (p4, M, 25, TH, LI)*

*We got introduced to these things by watching porn. Porn has taught us a lot and with porn we have gained knowledge and experience. (p10, M, 24, TH, LI)*

*Up to the age of 15 I used to believe the engagement ring is was the reason for pregnancy. I hadn’t asked anyone; it was my own conclusion. Imagine that .15! Then I was introduced to porn and I wanted to get into such relationships. Porn made me see stuff, the stuff that you haven’t ever seen or talked of in your life, and you don’t have any idea about, you haven’t seen a girl naked up to that age. (p10, M, 24, TH, LI)*

2iv: Books

*I just went and read the books that my mom had about pregnancy. (p1, F, 22, R, MI)*

*I read books. (p16, F, 25, AG, LI)*

*It was nothing in school, but my dad had bought me an oxford encyclopedia, I was around 13-14 years old. This book explained everything from A-Z, fertility and other stuff. So, when I learned about it, I went to school and explained it to my classmates. (p6, M, 25, AG, MI)*

*I’ve got the information I have now from books and the internet. As far as I know it’s the same with my friends. (p8, F, 25, AG, HI)*

*I started reading [about sexual health and relationships]. (p18, F, 18, AG, HI)*

*I would tell them to go and read some books. Now you can find reliable books on the internet. The other options is going to see a doctor. (p25, F, 25, R, LI)*

*I would tell him/her to search it on the internet or read a book about it. (p9, M, 22, TH, LI)*

2v: Personal or friend’s experience

*What we know is either from experience or searching on the internet. We have earned all the knowledge we have by ourselves. (p22, F, 25, AG, LI)*

*I started to understand it on my own or friends explained it to me. (p1, F, 22, R, MI)*

*It’s not perfect but the point is that most of the information they get is from their own experience, not from the education anywhere in school or university or anywhere else. They have to experience it to learn. I started experimenting. (p11, F, 25, TH, LI)*

*I believe nowadays the knowledge is increased due to exchange of information that kids do between themselves. This can interest others that have no idea what is going on and they’ll go and search further. This education spreads in a very personal level and the knowledge expands in more private groups. (p18, F, 18, AG, HI)*

*There wasn’t any sexual health education in school, anything I know now I’ve learnt from the internet or my friends. It all depends on how much you have researched on your own. (p22, F, 25, AG, LI)*

*I would tell them about my experiences. (p19, F, 24, TH, HI)*

*We just share our personal experiences with each other really. As none of us knows better to give advice to the others. (p10, M, 24, TH, LI)*

*I first would talk to them and advise them based on my own experience. (p21, M, 25, AG, HI)*

*A friend of mine once asked me this and I told her about my experiences. I told her about what I knew. (p22, F, 25, AG, LI)*

*It depends on the issue. Many of my friends come and tell me about their issues and I advise them based on my experiences, but I always remind them it worked for me, you should try it for yourself to see whether it works for you too. I wouldn’t ever say anything for sure. (p7, M, 21, TH, HI)*

2vi: Doctors

*Go see a doctor. (p1, F, 22, R, MI)*

*Go see a doctor. (p23, F, 20, AG, LI)*

*I would suggest her to go see my gynecologist. Because I trust her in her work. (p11, F, 25, TH, LI)*

*I would suggest her to go visit a gynecologist. (p12, F, 25, TH, MI)*

*I would suggest they go see a doctor. (p13, F, 25, TH, LI)*

*I would tell them to go and see a gynecologist. (p14, F, 25, R, LI)*

*If I don’t know about it, I would tell them to go see a doctor. (p14, F, 25, R, LI)*

*I would help them if I can, if not I would tell them to go see a doctor. (p15, F, 25, TH, LI)*

*If I don’t know anything about it, I would tell them to go see a doctor. (p16, F, 25, AG, LI)*

*I would tell them to go see a doctor. (p24, F, 22, AG, LI)*

*I’d tell them go visit a doctor, although it could be expensive, but it’s my only advice. (p8, F, 25, AG, HI)*

*I’d tell them to visit a doctor. (p2, F, 18, AG, HI)*

*Again, I’d tell them to visit a doctor. A good one, a doctor who wouldn’t ask her why she is pregnant while she’s not married, a doctor who doesn’t judge. (p2, F, 18, AG, HI)*

*I would tell them to go and see a Doctor. And if she/he has issues with her/his partner I would suggest them to talk and discuss it with their partner. (p5, F, 25, AG, MI)*

*I’d tell them to go and see a doctor. (p7, M, 21, TH, HI)*

*I would tell her to go visit a doctor. (p22, F, 25, AG, LI)*

*I would tell them to see a doctor. (p4, M, 25, TH, LI)*

**3. Availability and Quality / Content of Sexual Health Education**

Mainly in response to question 5 from the interview protocol.

5. What sexual health education have you received and who provided this?

1. Can you tell me about the topics that were included in this sexual health education?
2. How useful and comprehensive would you say this sex education was?
3. Are there any particular topics that you would have liked to have more coverage of?

*In my opinion there is nothing to be ashamed of. Some might say what are these shameful things that they have put in our books. But we should start learning this kind of stuff someday so what’s better than learning them in our schools rather than going and learning it from someone who doesn’t have the proper knowledge themselves. If we don’t teach them, they would go and experience it themselves. (p1, F, 22, R, MI)*

*In schools they don’t teach this kind of things. (p10, M, 24, TH, LI)*

*For me, the only person who could help a bit was my biology teacher however she wouldn’t get into details, I mean they couldn’t, as if they weren’t allowed to. I mean not only the teachers had boundaries to get into this kind of matters but also the students themselves couldn’t ask. (p3, F, 20, AG, HI)*

*…They used to teach a lot of stuff in it, but this module is completely removed from the curriculum now because they want to increase the population. But in that module, they talked about all pregnancy prevention methods. (p14, F, 25, R, LI)*

*Our educational system teaches us absolutely nothing about this kind of stuff. (p9, M, 22, TH, LI)*

*I think the new generation are more educated, and there’s no hope in our educational system. Maybe there were some teachers who told us some stuff, but it was really vague and useless. (p9, M, 22, TH, LI)*

*From the scale of 1 to 10 I would say 1. Because those information were really basic and I had known much more than that from the internet, but it was something new for some people. I even remember that a girl asked if she takes birth control pills would she become infertile forever and this was so weird for me because she used to have parallel and different relationships with different guys, and I thought to myself how is this possible? How can she be so illiterate? (p8, F, 25, AG, HI)*

*We’ve never had an official sex education class . They always try to keep kids away from this kind of topics and there are no resources for those who get into this kind of stuff. (p8, F, 25, AG, HI)*

*At the first year of high school in our biology class there were 2 pages about chromosomes and sperms, it was so vague that I can’t even recall what it was about. In university we had this module that didn’t have any books or other teaching materials. They provided us with 5 pages of information, briefly explaining birth control pills, IUD and tubectomy. Of course now all of these are omitted from the educational system and instead now they have come up with module about great values and meaningfulness of having a family and the foundations of it. Because there is this general policy to increase the population. (p8, F, 25, AG, HI)*

*If I wanted to get married with that level of knowledge, I would have been screwed. That’s zero information they give us. (p1, F, 22, R, MI)*

*Because it was only like, they just told us how to prevent pregnancy. (p11, F, 25, TH, LI)*

*In my opinion it's very low. Because we can’t get ourselves educated in official ways. In school and even in university, no education is given to us. We only have a 2 credited module in our universities called Family planning which I have personally passed it and my friends and people around my age have done it also but it's not enough at all and they don’t teach the necessary stuff. Young adults usually ask basic questions from each other that have very simple answers, but no one has ever given them the right answers. (p12, F, 25, TH, MI)*

*I went to a religious school. There were absolutely no teachings about sexual health. Even if someone wanted to get married had to leave the school because she might have found some sexual information and they were scared that it might get shared with others. (p12, F, 25, TH, MI)*

*2 /10. I would give 1 point to our lecturer who was a good lecturer however she only talked about women's general issues and not sexual issues. Mostly women’s social issues. Second point was for the prevention methods for unintended pregnancy that everyone should know about. (p12, F, 25, TH, MI)*

*I’ve learned everything I know through experience. No one has taught me anything. There were absolutely zero education. (p13, F, 25, TH, LI)*

*Nothing, I haven’t gone to university therefore I didn't take the family planning module as well. (p13, F, 25, TH, LI)*

*Except the Family planning module that we passed in university, which was not detailed enough, I would say nothing. I used others’ experiences when I started having sex. For my first sexual relationship I wasn’t taught anything from anyone, I had only read about it online. For the Family planning module lecturers only taught about the family issues but told us to read the parts which was about sexual stuff at home. They didn’t find it appropriate for mixed audience. (p14, F, 25, R, LI)*

*I would say 5 /10 and it is only for the pregnancy prevention methods that they taught us which they don’t anymore, it is because of the plan to increase the population. (p16, F, 25, AG, LI)*

*Nothing, in high school they just told us that after having sex or masturbation you should wash yourself in the way that religion approves, stuff like that. They only talked about the religious parts. So no education was given, and it’s never been talked about. (p17, M, 19, TH, HI)*

*There is absolutely nothing from school. (p18, F, 18, AG, HI)*

*Very little. Even nothing. After I already knew everything from my friends, my mom sat me down and talked to me about it. That’s all. (p19, F, 24, TH, HI)*

*Nowadays families give more information to their kids, but schools don’t give any education in this regard. (p19, F, 24, TH, HI)*

*In university there’s this module called Family sciences which I haven't taken it yet, but I’ve heard the content is things like love your husband and stuff like that. It’s not sexual health per se, it’s supposed to have that undertone to it. (p19, F, 24, TH, HI)*

*There wasn’t any sexual health education in school. (p2, F, 18, AG, HI)*

*1/10.Because it was only about how sperm goes inside the egg. That was all. (p2, F, 18, AG, HI)*

*Absolutely nothing. We never received any education about it because even talking about it was considered taboo. Parents also don’t discuss it. Best case scenario is that we search it online or a friend would tell us something, that’s all. Neither parents nor schools would teach us anything, nothing from educational system and not even university. Nothing was explained to us about sexual health. (p20, F, 23, AT, HI)*

*I took the new version. It seems that the previous version was about sexual health and stuff like that but not now. In the new version, they mostly talk about morality in the family. Things like the qualities that girls, boys or mothers and fathers should have. Stuff like that. There’s absolutely nothing about sexual health in it. Absolutely zero. It meant nothing. (p20, 23, F, AT, HI)*

*In university there was Family planning module but now it has been changed to Family sciences with very different subjects such as religious stuff with no mentioning of sexual health. They just say it is not a good idea at all to have sex before marriage or to speak of it! (p21, M, 25, AG, HI)*

*Nothing. Nothing in school. In university we had this module called Family planning which was absolute nonsense and didn’t have anything useful. (p22, F, 25, AG, LI)*

*We haven’t received any education however we have learned some stuff on our own. There wasn’t any education in school. (p23, F, 20, AG, LI)*

*We never have received that kind of education. It’s nothing in school. There was no education about this during school. We used to ask each other and there was no education from our teachers whatsoever. I haven’t passed the Family planning module because I never went to university. (p24, F, 22, AG, LI)*

*This is the only official education I’ve received. I was 21 years old when I took the Family planning module. This module is now completely changed because they want to increase the population. They have changed the name of the module to Family sciences with no trace of teachings about pregnancy prevention. Family planning was more about unintended pregnancies and ways to avoid it and STIs prevention but in Family sciences they only talk about STIs and sex really briefly and nothing about pregnancy prevention. (p25, F, 25, R, LI)*

*3/10 at best. I give it a 3 just for the basic stuff that they taught us. (p25, F, 25, R, LI)*

*I haven’t received any sexual health education of any kind. (p3, F, 20, AG, HI)*

*Anyone who got into university got to learn about this stuff. Now that they’ve changed the module, there is way less information available to students. If there are people who don’t attend university, they can benefit from brief explanatory classes that are held in health clinics before you get married and are compulsory. I think they only give a very brief explanation about sexual organs. You can’t say it is useful really. People who get married without having a university degree will be very uneducated and sexually illiterate specially if they don’t read and research on their own as they’ve never received enough education or any education at all for that matter. (p25, F, 25, R, LI)*

*In my opinion we live in a very conservative country and most people have a conservative perspective towards most issues so the only education kids get from their families would be if someone wants to touch you in a way you are uncomfortable with just run away and tell an adult about it. (p7, M, 21, TH, HI)*

*I attended a very religious secondary school, one day one of our teachers came and told us masturbating is really bad, he was so dogmatic that he told us masturbating equals killing future population. He never mentioned how babies are made and stuff like that, but he told us we have to wait, masturbation is not a good thing to do. It was really ambiguous. Then at university there was this module called Family Planning which wasn’t that bad, it was more about preventing unintended pregnancy rather than educating us about STIs. After all that, I might know about 20 to 30 percent of the whole subject. Things they teach in that module has completely changed because of current plans to increase the population. With all I said, how well educated do you think all of us are? Absolutely uneducated. (p6, M, 25, AG, MI)*

*It was nothing in school. (p6, M, 25, AG, MI)*

*Nothing really in school. In university we had this module called Family Planning and that was it which wasn’t enough. Current national policy is to increase the population and this module would contradict with that goal; therefore they have removed the module from current and future students’ curriculum. (p5, F, 25, AG, MI)*

*The thing is that we already knew the stuff that they taught us, I mean these classes weren’t really necessary because the students knew much more. People who had the experience of sexual relationships didn’t need to be told, they knew all that stuff. It was too late. (p5, F, 25, AG, MI)*

*Currently, when you want to get married, they send you to a 2-hour class to teach you this stuff, imagine only 2 weeks before you want to start your life as a couple. It is pathetic. You and your partner have done so many things sexually so far and 2 weeks to your marriage you would find out about things you used to do wrong. It is too late. (p4, M, 25, TH, LI)*

*We all learned about our differences in sexual organs, while pretending to be doctors and patients when we were kids. It was a game that helped us figure things out. We didn’t get any education. (p4, M, 25, TH, LI)*

*Before getting into university and having a 2-credit module about family planning which is really vague, there is no education at all. (p4, M, 25, TH, LI)*

**4. Understanding and Negotiation of Sexual Relationships**

Mainly in response to question 8, 9 and 13 from the interview protocol.

8. How confident do you feel that you can prevent unwanted pregnancy or STI in your present or future sexual relationships?

9. Do you think sexually active young adults are in Tehran are protecting themselves against sexually transmitted infections?

1. If so, how?

13. How confident and in control do you think a typical 18–25-year-old Tehranian is in managing sexual relationships?

4i: Familiarity implies health in sexual partners

*I got vaccinated for HPV but before that I’ve had unprotected sex with someone whom I really trusted and didn’t even think that he might’ve had unsafe sex before me. After that, all my sexual activities were protected ones. (p8, F, 25, AG, HI)*

*I’ve never had a partner before this guy, but he told me he’s been with 3 or 4 girls before. I prefer to believe that those girls were all healthy. (p25, F, 25, R, LI)*

*We didn’t get tested together because we didn’t see it necessary. I think I believed that my partner is healthy. I decided to trust him. I think we will never get ourselves tested and I would carry on with this trust unless I see a symptom on his body. (p25, F, 25, R, LI)*

*I know him, that’s why I have sex with him. I know him through the connections we’ve had. (p24, F, 22, AG, LI)*

*Time and duration that you know the person could be an insuring factor. For instance you know someone for 10 years and you are sure that she has only been with you during this period. There’s no guaranty, it all depends on the trust that exists between the partners. (p21, M, 25, AG, HI)*

*It rarely happens that someone becomes concerned about STIs because they trust their partners and believe that they haven’t been with unhealthy people. They would say I’ve known this person for such a long period as if longevity and duration of knowing someone before sleeping with them is a guarantee of them being sexually healthy. It’s stupid I know, but almost everyone is like that. (p14, F, 25, R, LI)*

*50 percent of youth are protecting themselves and the rest are having unprotected sex. I don’t think it is costly at all. It is more because of lack of knowledge and false trust. They believe whoever they are sleeping with is definitely healthy and they don’t believe it can happen to them too. I used to be like that not so long ago, I used to be confident of my partners health but now I have doubts and protect myself. (p13, F, 25, TH, LI)*

4ii: Confidence and power in managing sexual relationships

*I can protect myself, but it doesn’t mean that I wouldn’t be worried about things, but I have enough information and I have enough experience to deal with these situations. (p11, F, 25, TH, LI)*

*I have never had any concerns about it because I think I know how to protect myself. I believe I know how to take care of myself. (p15, F, 25, TH, LI)*

*If someone tries to force me into having sex with them, I can always defend myself and get over my shyness. Because If I actually am honest to myself, I know that there are so many people who want to have sex and I don't have to give in to just anyone for the sake of having sex. Therefore I think I can 100% manage my sex life. (p17, M, 19, TH, HI)*

*I can only manage it to an extent that if my partner gets pregnant, I know how to abort the child. We don’t know how to properly manage a sexual relationship at all. (p4, M, 25, TH, LI)*

*In my opinion sex is related to confidence in so many ways. I mean it depends on how attractive or lovable you think you are. The stronger this feeling the more you can manage your relationships and the less you have this feeling, chances are you mess it up more and you can’t manage it properly. I think I’m not that confident yet to manage my relationships properly. (p5, F, 25, AG, MI)*

4iii: Communication in Sexual Relationships

*It all depends on their relationship and their partners. If they are able to discuss and talk about everything and be comfortable with each other, everything can be solved and managed. For example myself, I always wanted to talk about STIs with my partners, but I was always scared of them telling me I was accusing them of being ill, so I never told them that I was concerned about STIs. I only said let’s use condoms because I am scared of getting pregnant, so I always hid my concerns about STIs. (p8, F, 25, AG, HI)*

*I usually try to talk about this kind of stuff before starting any relationship and would tell him about what I want before sex. (p14, F, 25, R, LI)*

**5. Concerns about Sexually Transmitted Infections (STIs)**

Mainly in response to question 4 and 9 from the interview protocol.

4. How concerned do you think young adults are in Tehran are about unwanted pregnancy and/or sexually transmitted infections?

9. Do you think sexually active young adults are in Tehran are protecting themselves against sexually transmitted infections?

1. If so, how?

5i: Ambiguity and lack of education on STIs

*Because they haven't been warned enough about it although there was a period that HIV became a hot topic, however it was really vague due to the conservative nature of our society. Even in university it is vague, they only come and give out names of some STIs. I mean people are not concerned because they don't know about it. (p25, F, 25, R, LI)*

*There are STIs out there that I may not know about, but pregnancy is a straightforward process to understand and to avoid. (p8, F, 25, AG, HI)*

*I am mostly concerned about the STIs. Because I’ve seen it in people around me, not a specific disease but I’ve seen friends of mine with different infections. They have gotten UTI, and they were boys. I believe UTI mostly comes from having sex. (p21, M, 25, AG, HI)*

*We haven't been educated for it and this can be as harmful as the diseases itself. We don't consider STIs a serious disease. We need to learn about them. (p9, M, 22, TH, LI)*

*About STIs because they are getting more various, they are causing more concerns so I’m being more careful. For example I hadn't heard about HPV before, but I’ve learned about what it is recently. About Hepatitis for instance I’m really worried about how it gets contracted. My biggest concerns are the common sexual diseases. (p13, F, 25, TH, LI)*

5ii: Invisibility of STIs

*The fact that you can hide your STI from others makes them not to be concerned about it. (p12, F, 25, TH, MI)*

*…These diseases don't show visible symptoms so no one would find out about it, for example HPV, no one would find out about it most probably. (p12, F, 25, TH, MI)*

*You know STIs is something that you get, and you are the only one who is going to know and is gonna be bothered. (p11, F, 25, TH, LI)*

5iii: Fear and worry about STIs

*I have never had any concerns about it (STIs) because I think I know how to protect myself. I believe I know how to take care of myself. (p15, F, 25, TH, LI)*

*The majority aren't concerned about STIs. (p14, F, 25, R, LI)*

*I’m really concerned about STIs to the extent it kills the joy of having sex. I’m more scared of HIV, because they have presented it as this big and scary disease that ends up killing you. (p17, M, 19, TH, HI)*

*I am really afraid of STIs. (p16, F, 25, AG, LI)*

*STIs concern me the most. You know if you give your STI to someone else you might change the course of their lives forever. (p4, M, 25, TH, LI)*

*I get worried because you can’t tell if your partner is healthy or not. (p16, F, 25, AG, LI)*

*To be honest I haven't thought about pregnancy so I haven't been concerned about it but for STIs I would say yes. I usually try to look for visible symptoms in my partners to see if they have STIs that have visible symptoms such as HPV. I am concerned about STIs but not for unintended pregnancy. (p14, F, 25, R, LI)*

*…You can abort the baby, but HIV will stay with you forever. (p17, M, 19, TH, HI)*

*My other partners always insisted on not using a condom, but I never accepted. Mostly because of the fear of HPV and AIDS. I’m generally concerned about the STIs. 100% of women are concerned about unintended pregnancy, but only 10% are concerned about the STIs. (p8, F, 25, AG, HI)*

*I am personally more concerned about the STIs. (p18, F, 18, AG, HI)*

5iv: Perception of other groups’ lack of concern

*People around me usually get concerned when something happens to them. However it rarely happens that they become concerned to the point that they want to protect themselves or use preventive methods to avoid any issues. (p14, F, 25, R, LI)*

*Some guys are careless and don’t use any kind of protection, they just want to have sex. (p18, F, 18, AG, HI)*

*Guys will be very careless most of the times and they are like: Who’s got time and energy for such stuff. There might be so many people who can afford it financially but then that carelessness stops them from seeking medical attention. (p21, M, 25, AG, HI)*

*I believe people who are younger than us are more careless in their sexual relationships and people around my age are more careful. (p8, F, 25, AG, HI)*

**6. Concerns about Pregnancy**

Mainly in response to question 4 the interview protocol.

4. How concerned do you think young adults are in Tehran are about unwanted pregnancy and/or sexually transmitted infections?

6i: Fear and worry about pregnancy outside marriage

*There is no concern about unintended pregnancy, there are thousands of ways to prevent it. Even if it happens again there are thousands of ways to fix it. I have absolutely zero concerns. (p13, F, 25, TH, LI)*

*Others are really stressed about getting pregnant although you can simply prevent it or you can solve it afterwards, there’s nothing to worry about. (p13, F, 25, TH, LI)*

*Unintended pregnancy is not that important to me. (p4, M, 25, TH, LI)*

*They are concerned about pregnancy because they know about it. (p25, F, 25, R, LI)*

*Unintended pregnancy is a different story, because of the laws of our country it has become a great concern for everyone. I see lots of people who are worried about pregnancy after having a sexual relationship. Because here you should put your names on the paper and get married legally before doing anything. If you don't find out and the pregnancy gets to the stages that you can do nothing about it, either some would seek abortion which is not legal, or they are forced to get married which has got a hefty price. You're making a pricey decision by force without even being ready for it. A gunshot wedding they call it! That’s why there is so much concern about unintended pregnancy, it is so much that it surpasses the worries for STIs, so no one would even think about STIs at all. (p10, M, 24, TH, LI)*

*Because in Iran, it is illegal for people to have sexual relationships before getting married. It is something everyone does, but you would be blamed for it. Becoming pregnant out of marriage is way worse, people are getting more open minded but still there are many dogmatic people out there. Unintended pregnancy is definitely much worse because STIs might be treated but you can’t “treat” pregnancy. (p2, F, 18, AG, HI)*

*I am concerned about pregnancy, because I don't believe in common prevention methods and I use natural methods. Natural method means pulling out before ejaculation. I usually do this. (p16, F, 25, AG, LI)*

*I guess I’m more scared of pregnancy. (p11, F, 25, TH, LI)*

*I’ve had friends who had the phobia of becoming pregnant so they couldn't really enjoy having sex even while wearing condoms or using other preventive methods. (p14, F, 25, R, LI)*

*For heterosexuals, the unintended pregnancy is more of an issue. It doesn't only concern girls but also boys are concerned, I mean boys also freak out about what to do and how to fix the mess. (p18, F, 18, AG, HI)*

*In my opinion this concern is more towards unintended pregnancy. Not for me but for people around me. They are more scared of pregnancy. (p19, F, 24, TH, HI)*

*I am concerned about unintended pregnancy, obviously it isn't my fault if it happens. My partner should be more responsible and pull out on time. (p20, F, 23, AT, HI)*

*99% of those who have sex are concerned about unintended pregnancy. (p25, F, 25, R, LI)*

*In my case I’m really scared of unintended pregnancy and I carry this fear with me every single time I have sex. (p5, F, 25, AG, MI)*

*They're mostly scared of unintended pregnancy. (p9, M, 22, TH, LI)*

6ii: Visibility of pregnancy leading to social and personal issues

*I think some people are more concerned about unintended pregnancy because of their social image. (p17, M, 19, TH, HI)*

*If you get pregnant the whole world would find out after 9 months. Even after 4 months everyone will notice so they see that the consequences of getting pregnant is so much more, it’s even same for boys, they think if my partner is pregnant, she would definitely expect me to marry her, or I would get into trouble or I need to pay for the abortion or even the struggle of thinking about keeping the baby or not or our relationship might be damaged and so many thoughts like that. (p12, F, 25, TH, MI)*

*I remember that it was around 1 or 2 years ago that we were in a gathering and one of my closest friends came by and she was like I am 100% sure that I am pregnant, and we were all scared as hell, not because of the pregnancy itself but because of the consequences. Therefore I believe probably everyone is concerned. The social judgment that comes with those things ruins your life for good. (p18, F, 18, AG, HI)*

*I know that concerns are much bigger for unintended pregnancy because Umm, pregnancy has some consequences, first and foremost would be getting excluded from the family. Secondly you have to raise a child which obviously doing so independently in Iran is really difficult especially for women. (p3, F, 20, AG, HI)*

*Pregnancy is the main concern because they are worried about their reputation and what would society think of them. (p4, M, 25, TH, LI)*

*If there is any concern at all, it’s for unintended pregnancy and the main reason is families being traditional and conservative. The second reason is the endless expenses that comes with it. (p7, M, 21, TH, HI)*

**7. Contraception and Condoms**

Mainly in response to question 15 from the interview protocol.

15. What contraception methods do you or your partner currently use or plan to use in your future relationships? Why?

*[I] use natural methods, Natural method means pulling out before ejaculation. (p16, F, 25, AG, LI)*

*Male condoms, because in addition to contraception it prevents STIs. (p9, M, 22, TH, LI)*

*Condoms, but we don't always use it while having oral sex. It depends on the situation. Actually you can’t rely on withdrawal. One of my coworkers was pregnant for the second time so I asked her why you don’t use protection, why don't you take pills. Then I found out they use withdrawal so I’m not sure if it’s a good method after all. (p24, F, 22, AG, LI)*

*Condoms and contraceptive pills. (p18, F, 18, AG, HI)*

*Just regular condoms. Because I trust them and know more about them than the other methods. (p19, F, 24, TH, HI)*

*Male condoms and pills at the same time because prevention is better than looking for treatment. (p2, F, 18, AG, HI)*

*Just condoms because it seems pills are a bit problematic for me. (p20, F, 23, AT, HI)*

*I use condoms and if not, I am really careful. (p21, M, 25, AG, HI)*

*The thing is I know many of them wouldn’t use it. For instance, this person has sexual relationship and when you ask if they use condoms, she would say no he just pulls out at the last moment and ejaculates outside, then I would say do you know that pre-ejaculatory fluid could …. She would say no, no, no it’s totally fine. I know that at the heat of the moment many people would neglect. (p3, F, 20, AG, HI)*

*You can find pills everywhere. (p10, M, 24, TH, LI)*

*Contraceptive pills and stuff like that can be easily found, I don't know about the prices I just know they can be easily found and are accessible. (p18, F, 18, AG, HI)*

7i: Condom availability and accessibility

*Condoms, because of accessibility. (p1, F, 22, R, MI)*

*Condoms because it is the most accessible method. (p14, F, 25, R, LI)*

*It is accessible everywhere. Condoms are everywhere and can be easily purchased. I don't think Iran makes condoms. Condoms can be found everywhere but the quality might not be that good depending on the social economy of that neighborhood you're shopping in. (p10, M, 24, TH, LI)*

*I think condoms can be found in every pharmacy. And definitely in deprived areas there are pharmacies that provide different brands of condoms. So condoms are accessible everywhere. (p12, F, 25, TH, MI)*

*I don’t think the accessibility depends on districts that you live in. It all depends on how much the person cares. Because there are people who don't earn much however they really care about their sexual health and go see doctors and things like that. (p14, F, 25, R, LI)*

*Everything is accessible everywhere. (p15, F, 25, TH, LI)*

*You can easily find condoms everywhere. I don't know much about the quality. I think it is accessible. (p17, M, 19, TH, HI)*

*I don't know about good doctors, but condoms are accessible everywhere. (p19, F, 24, TH, HI)*

*I don't know about pills and doctors. In deprived areas the culture is a bit different, and they are more conservative, so I don't know if they go after it or not. I mean they are accessible, so it depends on the person and whether he wants to get it or not. (p19, F, 24, TH, HI)*

*I think it is available for everyone. (p22, F, 25, AG, LI)*

*It is accessible everywhere. You can find it in both pharmacies and supermarkets. Therefore the accessibility and availability are good. (p22, F, 25, AG, LI)*

*Thank god you can find it everywhere. You can even find it in supermarkets now. Condoms are everywhere, I mean it can be found in all districts. (p25, F, 25, R, LI)*

*Condoms are available everywhere, even in supermarkets. You can find condoms. (p3, F, 20, AG, HI)*

*Iran doesn't produce condoms, but you can almost find everything you need. (p6, M, 25, AG, MI)*

*I don't think it's accessible. (p11, F, 25, TH, LI)*

*I think the only place that condoms, foreign condoms can be bought are the pharmacies or supermarkets. (p9, M, 22, TH, LI)*

7ii: Condom cost

*They are expensive. I think if a middle-class couple want to have regular sex, for example 2 or 3 times a week, this would be a heavy cost for them, they might be able to handle it, although hardly, but anyone from lower class families can’t. Birth control pills are cheaper but, in my opinion, they are the worst for women, overall the contraceptive methods get more limited as the socioeconomics go lower. I believe only women from working class backgrounds use pills. Especially as people from more deprived areas usually have lower levels of education and therefore it is common that men who are selfish say they don't want to use condoms because they don't like it and women have no option but to accept to take pills to prevent pregnancy because they can’t afford other methods. (p8, F, 25, AG, HI)*

*I don’t think that costs are cheap or reasonable. There is no Iranian produced condom. Condoms are around 170000 Rails per pack. I don’t know about the price of pills because I don’t purchase them. Maybe I can afford this cost but for someone from a deprived area, or someone who works as a pizza delivery guy, can’t afford it and would not pay such prices as they think it’s not worth it. (p4, M, 25, TH, LI)*

*The price is average. Though not everyone can afford i.e. think the price of condoms should be around 300000 to 3500000 Rials per pack. Nowadays people in Iran are facing various financial difficulties and also many people have other priorities. Some people prefer to buy new clothing rather than paying for their sexual health. (p3, F, 20, AG, HI)*

*I think they are expensive. For foreign condoms as imports are getting more complicated due to sanctions the prices are getting higher so they are more expensive specifically for poorer people, we can see that they have more children as the result, maybe lots of them are results of unintended pregnancy. (p20, F, 23, AT, HI)*

*It is not cheap unless it has a bad quality. If you want something with high quality, you have to pay a lot. For instance condoms, a package with 3 condoms in it is around 210000 Rials. (p16, F, 25, AG, LI)*

*Condoms are relatively cheap but it’s not the same for visiting doctors. (p19, F, 24, TH, HI)*

*I think the majority can afford it, but they think it’s not worth it, personally I believe It is worth it and I would spend my money on it. (p3, F, 20, AG, HI)*

*The cost is reasonable. The last time I bought condoms which was one year ago a pack with 6 or 8 condoms was 150000 Rials. (p24, F, 22, AG, LI)*

*It’s reasonable. Condom packs are around 300000 Rials with 6 condoms inside. They are not expensive. Working class people can definitely afford it however there are people below that level, people who are poor or jobless. These people have unprotected sex. If this is what you mean by inexpensive, it is not for everyone, because in my perspective inexpensive means something the majority can afford. It doesn’t include poor people. In my opinion it doesn’t include jobless people either because they can’t afford it anyway. Other necessary things that they can’t afford are food and clothes. Therefore when they can’t afford such basic things they don’t fit into my statistical range. It is affordable and reasonable for people who can afford basic stuff. (p23, F, 20, AG, LI)*

*I don’t know about the prices, but I think they are reasonable. (p22, F, 25, AG, LI)*

*I guess condoms are just fine. 3 in a pack sells for something like 60-200 thousand Rials. It’s only the brand that is different. I personally buy “Good Life” for around 400000 Rials. Therefore everyone from every socio-economic background can at least get the Iranian or Chinese ones. I understand that they are not good brands but at least they can protect to an extent, probably. (p21, M, 25, AG, HI)*

*It’s neither expensive nor cheap. Condoms are 300000 Rials per packages which contains 4 to 4 condoms. It’s regular. (p15,F,25,TH,* *LI)*

*It is something regular, everyone can purchase it, but I don’t know the exact price. (p13, F, 25, TH, LI)*

*Condoms are relatively cheap. (p19, F, 24, TH, HI)*

*Iranian condoms are really cheap, I think a package with 12 condoms costs only 60000 Rials. (p12, F, 25, TH, MI)*

7iii: Quality of Iranian condoms

*The Iranian ones that are sold for 60000 Rials are not good at all. (p21, M, 25, AG, HI)*

*From what I’ve heard Iranian produced condoms are not reliable at all. (p20, F, 23, AT, HI)*

*Iranian condoms are accessible for everyone but lack quality and tear apart easily. (p8, F, 25, AG, HI)*

*I haven't ever seen Iranian condoms. The current policy of increasing the population is in contrast to these things so why would we produce it? (p9, M, 22, TH, LI)*

7iv: Ease of use

On contraceptive of choice

*I guess the easiest and cheapest are condoms which I have always used. (p11, F, 25, TH, LI)*

*Condoms because it’s easy to use and you can find it anywhere. (p12, F, 25, TH, MI)*

*Condoms, it is the easiest and most accessible method. (p13, F, 25, TH, LI)*

*Condoms and pills because they are easy to use. (p15, F, 25, TH, LI)*

*Contraceptive pills and condoms. Because they're easily available. (p18, F, 18, AG, HI)*

*Condoms and pills because they are easy to access. (p23, F, 20, AG, LI)*

*The easiest way is to use condoms however I really wanted to use an IUD but when I went and saw my gynecologist, she told me they only do it for women who have given birth. (p25, F, 25, R, LI)*

*Withdrawal, Condom, natural cycle. Because of convenience. (p3, F, 20, AG, HI)*

*Condoms. They’re easy to find and use. (p4, M, 25, TH, LI)*

*Male Condoms. Because of accessibility and ease of use. I’d actually wanna try female condoms to see how they feel. But I’m not going to use pills. (p5, F, 25, AG, MI)*

*Condoms or pills. Ease of use and accessibility. (p6, M, 25, AG, MI)*

*I don't usually use any but if I do, I would go for male condoms and that’s for accessibility and convenience. (p7, M, 21, TH, HI)*

*Male condoms only because it's the most accessible, easiest and cheapest method. If I get in a serious relationship with someone, I would ask him to undergo tubectomy. Maybe I would try IUD one day, but I will never take birth control pills for sure. They mess up your entire system. (p8, F, 25, AG, HI)*

7v: Inconsistent use

*I know so many people among my friends that condoms and contraceptive pills mean nothing to them. (p18, F, 18, AG, HI)*

*All the people that I know have unprotected sex. (p17, M, 19, TH, HI)*

*I had this friend whom I told just go and buy it [condoms] but he refused. He said I don’t like it, and nothing has happened, and nothing will happen to me, which is a wrong attitude. (p21, M, 25, AG, HI)*

*Using condoms is always a thing that others advise me about, but I’ve always refused. (p10, M, 24, TH, LI)*

*I don’t like using condoms. It doesn’t give the real touching sensation; it is like you are putting it in a plastic. I prefer to be sure of myself and my partner. I mean to be sure that neither of us have any diseases. (p10, M, 24, TH, LI)*

*In my opinion you’re better off not using condoms because you might be risking with a low quality one, maybe this way you would pull out because you don’t have that trust. Still unintended pregnancy might happen, you would never know. Better to put that trust in yourself rather than piece of plastic. (p10, M, 24, TH, LI)*

**8. Barriers to Using Sexual Health Services**

Mainly in response to questions 16 and 17 from the interview protocol.

16. How accessible do you find contraceptive methods and sexual healthcare for young adults in Tehran?

17. Can you think of any reasons that young adults would avoid seeking sexual health knowledge and/or sexual healthcare in Tehran?

*No I don't think there are such things actually as sexual health clinics. (p10, M, 24, TH, LI)*

*No, I haven't ever heard of those [sexual health clinics]. (p2, F, 18, AG, HI)*

*I’ve never heard of such clinics and if there are any, there is no advertisement for them. (p4, M, 25, TH, LI)*

*I haven't heard of such places [sexual health clinics]. (p5, F, 25, AG, MI)*

*No not that I know of [sexual health clinics]. (p6, M, 25, AG, MI)*

*I don't know any sexual health clinics in Tehran though, so maybe for people like me, who have the money to spend, a part of it is the lack of information on where to go and who to trust to spend their money on. (p7, M, 21, TH, HI)*

*I think there are some sexual health centers that are supervised by either community centers or ministry of health, I don't know exactly but I’ve heard you can get tested for HIV for free. I haven't seen any adverts or general information about it though because it's not something that is meant for public knowledge. It’s kind of a hidden service, because of sex being a taboo. (p8, F, 25, AG, HI)*

*So many don't know that sexual health clinics exist, for example the centers that I told you before that test for HIV for free, I heard about them from a friend, I haven't seen any advertisements or public notices about them in the city, magazines or newspapers, not even a billboard. (p8, F, 25, AG, HI)*

*I’ve heard of some sexual health clinics, but I’ve heard they only serve married couples. I’ve seen some kiosks recently that inform people about AIDS. There was one in front of the subway station. In the Family Sciences module they tell us that we should refer to these clinics, but where are these places and why we don't see any advertisements about them? We don't see anything about it neither on TV nor on billboards. If they exist why do they hide it from us? (p9, M, 22, TH, LI)*

*We don't know where the sexual health clinics are. (p9, M, 22, TH, LI)*

*All good doctors are accessible in all areas of the city. (p22, F, 25, AG, LI)*

8i: Cost of visiting doctors and sexual health care

*Even I might avoid going to see the doctor if it cost me a lot. (p16, F, 25, AG, LI)*

*I have so many questions which I don't have the answers to, and I can’t afford to visit a doctor to ask them. (p8, F, 25, AG, HI)*

*Poverty is a real issue. In below the average families, mothers constantly sacrifice themselves. They prioritize every cost of the house over their health. This is really bad, that’s why we see that so many pre and post pregnancy tests don't get done in such families. So many cancer patients could have been treated and cured but unfortunately, they get to it when it is too late. (p25, F, 25, R, LI)*

*[Naming barriers in seeking sexual healthcare] Expenses, embarrassment, seeing disease as permanent disability and damage. Sexual stuff being a taboo. (p6, M, 25, AG, MI)*

*It all depends on the social economic situation of each individual. Imagine a manual worker, no matter how hard he works his income won’t ever be enough for his day-to-day expenses, let alone the expenses for sexual healthcare. But in my opinion people should save a part of their income for this purpose just like they do for their cardiologists. (p7, M, 21, TH, HI)*

*One reason is this and the other one is doctors expensive cost that make so many people forget about going and seeing a doctor, they would say they rather search it on the internet on their own. (p20, F, 23, AT, HI)*

*It could be for various reasons. First is that most of us are financially dependent on our families and we don't want to spend all that pocket money which is not much on visiting doctors so we prefer to go with our parents so that they would pay. (p2, F, 18, AG, HI)*

*I would pay anything because it is really important to me. It is expensive though. (p6, M, 25, AG, MI)*

*I guess the right way is to get tested for STIs at least once a year, but I don’t do it because it is so expensive for me. (p4, M, 25, TH, LI)*

*It is expensive but some would pay for it. Those who are rich and care about their sexual health. Most people don’t care but it should be cheap so that everyone can benefit from it. (p2, F, 18, AG, HI)*

*Right now it is expensive, but it would be better to be cheaper and more reasonably priced so that everyone can afford it. (p17, M, 19, TH, HI)*

*As a whole, sexual healthcare including tests for detecting STIs and doctor visits are expensive. (p17, M, 19, TH, HI)*

*It’s not like it’s that expensive that no one can afford it. However it might not be really affordable for everyone to visit a doctor a few times a year. (p14, F, 25, R, LI)*

*Actually it’s as high as other kinds of medical care you can have, it’s not very expensive but it’s not also reasonable. The problem here is that if a person wants to afford all these, maybe he or she doesn’t have any income so if you don’t have any income it’s going to be hard for you to afford that. Especially in the 18–25-year-old age range. (p11, F, 25, TH, LI)*

*The HPV vaccine for instance, I paid around 10 million Rials for it. Middle class people can’t afford this amount easily and lower-class people can’t afford it at all. The cost of getting tested for HPV is around 3 to 4 million Rials. When they can’t even afford getting tested, they subsequently forget about the treatment, even when they test comes positive for STIs. The cost is the biggest obstacle. The cost of visiting a specialist doctor is 600000 Rials. How many times can someone afford that? Imagine someone who is trying for pregnancy or someone who needs treatment. It’s a hefty cost. (p8, F, 25, AG, HI)*

*The cost of a single visit for some doctors is really high. (p19, F, 24, TH, HI)*

*Also the cost of treatment is not cheap at all. For example, a simple test for STIs can cost around 2000000 Rials. (p16, F, 25, AG, LI)*

*The costs are almost reasonable. (p25, F, 25, R, LI)*

8ii: Trust in doctors

*Doctors are not trustworthy. (p4, M, 25, TH, LI)*

*We are scared to tell the doctor about our issues, for example to tell them we've had sex out of marriage, and they would let our families know about it. I’m absolutely terrified about that. (p2, F, 18, AG, HI)*

*Actually the problem I had with my gynecologist; she would say you are too young to have sex. I was 22 then. I was like my mother had me when she was 22. I’m not getting bothered with sex, I am enjoying it. (p11, F, 25, TH, LI)*

*Maybe it is the attitude of the doctors. Because I have this experience with a few gynecologists, they treated me terribly and were like why you had sex out of marriage and stuff like that. Having an experience with a doctor like this would make you think that everyone would treat you the same. The doctor that I used to go to was really judgmental, when she found out that I wasn't married and I was sexually active, she examined me with hate and didn't properly answer my questions. (p20, F, 23, AT, HI)*

*The fear of being blamed and judged by the doctors, why did it happen? Why were you careless? Why have you done this? (p14, F, 25, R, LI)*

*Then going to the doctor with parents is really scary as you are worried about what the doctor would tell them. (p2, F, 18, AG, HI)*

*We have always been distrustful of everyone, teachers would always turn us in to the principal’s office, anything we ever told our mom she would tell dad, that’s how we've become so cynical of everyone and everything. (p2, F, 18, AG, HI)*

8iii: Embarrassment as a barrier to sexual protection

*I think they are embarrassed to go to the pharmacy and ask for condoms, especially boys, I’ve seen it among my friends. (p19, F, 24, TH, HI)*

*They are also embarrassed to go buy condoms. (p6, M, 25, AG, MI)*

*The problem is me myself or the majority of people I know, regardless of their gender, feel embarrassed to go and ask for it in their own neighborhood, or even other neighborhoods as they are scared to get caught red handed by someone they know. For pills it's easier but pregnancy home tests are the same as condoms. You would think what the other person next to you would think about you, what if they were an acquaintance who would tell your father. When I was younger, I was really embarrassed to ask the pharmacist for condoms. But from a point onwards I told myself it’s their job to sell those things but still I am afraid of running into someone I know. It is really awkward for me to ask for it if there is anyone else around, so I prefer not to buy the condoms myself. This situation is even worse in more deprived areas because of different social norms. (p3, F, 20, AG, HI)*

*Some people are embarrassed to go and ask for condoms in a pharmacy because it’s usually out of hand reach and you should ask someone to give it to you. If it’s a lady selling it, it’s even worse for men, they would be even more embarrassed. Again if there is a huge age difference and the lady is so young, men would be further embarrassed. (p21, M, 25, AG, HI)*

*I guess people are embarrassed to go and ask for it in pharmacies, it might be one of the main reasons that they don’t use it. For example they are embarrassed to go and ask for lubricants or condoms. (p22, F, 25, AG, LI)*

8iv: Taboo shame and social disapproval as barriers

*[On what would stop them from visiting a doctor] Sexual stuff being a taboo. (p6, M, 25, AG, MI)*

*I believe it's the cultural background. They are like we don't have sex out of marriage and it's a very sensitive issue. It is a taboo so you can’t tell people I had sex and because of that something has happened to me and now I need to go see a doctor. Or even nothing has happened, and I want to go see a doctor for my sexual health. This is not really common. Therefore I believe first is the fear that we have of the judgment we get and second is the taboo which hasn't been broken yet and above all is the worry of what people might think and judge. (p18, F, 18, AG, HI)*

*The embarrassment of being judged for having sex out of marriage. For example they would be embarrassed or scared of their reputation to say they have gotten an STI. I don't think people would think that the doctor would call the cops, although they could and there is law enforcement in place for sex out of marriage, but they are scared that the doctor might call and tell their parents about it. For example, my mom suggested to go a doctor that is a friend of my aunt, but I refused. I searched and found another doctor. Because my mom is really sensitive about our family’s reputation and at that time, she didn't know that I was having sex out of marriage, that’s why I didn't go to that doctor to avoid any consequences. (p19, F, 24, TH, HI)*

*In my opinion the fear of bringing it up is the main reason. If the person has sex out of marriage, they would be afraid that others might find out specially if they are a female. But if they're ok with it (others knowing of their out of marriage relationship), then I can’t see why they wouldn't visit a doctor. (p5, F, 25, AG, MI)*

*You could be scared of being judged. About your health, about your experiences. (p11, F, 25, TH, LI)*

*I think it’s the fear of getting judged by the others. What others might think of me if they find out that I have sex out of marriage. I know so many guys that believe that their girlfriends are not decent and good people because they sleep with them. If she’s a good girl, why did she have sex with me? These backward, weird conservative way of thinking has roots in our male dominant society which put our women under so much pressure that she can’t find the courage even at the age of 30 to say I’m independent and this is my personal territory and not even my parents have the right to talk about my personal life. (p12, F, 25, TH, MI)*

*Because having sex here is a much-hidden thing. You wouldn't go and visit a doctor. (p10, M, 24, TH, LI)*

*Maybe they are embarrassed to go and get naked in front of the doctor and get checked up. (p13, F, 25, TH, LI)*

*Many people hate the special bed that gynecologists use, the one that you should bring up your legs to. Or they are embarrassed. (p22, F, 25, AG, LI)*

*The embarrassment of getting naked in front of a stranger should be the reason. (p24, F, 22, AG, LI)*

*The first reason is the embarrassment. People feel embarrassed to reveal their personal and private life to the doctor. (p2, F, 18, AG, HI)*

*The first problem is that ridiculous shame that we Iranians have. It seems we get annoyed by going to a doctor and telling them I have this or that pain. Why would we immediately go and see a doctor if we feel pain in our heart but when we have sexual problems we wouldn’t? (p9, M, 22, TH, LI)*

8v: Health motivation

*They think it only happens to others and they are always safe. They think it's not possible to get a virus, so they never concern themselves about it. (p12, F, 25, TH, MI)*

*Umm, about the STIs from what I know people generally aren't concerned about their health until something happens to them. (p3, F, 20, AG, HI)*

*[On what would stop them from visiting a doctor] Carelessness. (p14, F, 25, R, LI)*

*The only reason that comes to my mind is carelessness or laziness. Otherwise there’s no embarrassment or things like that. If I need to go to the doctor I would do it for sure, the only thing that stops me from doing that is laziness. For other women it depends on their age. Current generation are not embarrassed at all. Our main issue is carelessness and laziness. I don't think we are scared or ashamed or anything. (p23, F, 20, AG, LI)*

*Sometimes it is because of carelessness though. Some people simply don't care. (p16, F, 25, AG, LI)*

*Maybe it’s not important to them or they neglect it. (p13, F, 25, TH, LI)*

*Not prioritizing this issue. Not caring enough about it. (p10, M, 24, TH, LI)*

*There are some people who don’t care at all. (p18, F, 18, AG, HI)*

*There are some who care and protect themselves, on the other hand some people don’t care at all; these are in the majority. In my opinion young adults who are educated care more about protecting themselves. Obviously, those who are from poor families don’t care much. (p9, M, 22, TH, LI)*

8vi: Denial / fear

*If there’s anything in my mind that would stop me from doing it, is running away from problems. I mean I don't want to know that I’m sick, but I have to do it anyway. I think you get stressed after having unprotected sex. It scares you. So the main reason that stops me from going to visit a doctor is the fear of knowing that I have an illness. That should be the same with others too. (p17, M, 19, TH, HI)*

*[On what would stop them from visiting a doctor] Seeing disease as permanent disability and damage. (p6, M, 25, AG, MI)*

*Knowing you are ill is scary to some and so many people don't want to face the harsh truth. (p8, F, 25, AG, HI)*

*So many people don't care about STIs or pretend that they don't exist. (p19, F, 24, TH, HI)*

**9. Sexual Prohibition**

Mainly in response to question 17 from the interview protocol.

17. Can you think of any reasons that young adults would avoid seeking sexual health knowledge and/or sexual healthcare in Tehran?

*There are concerns, I mean the fear of getting into trouble. Other than the problem itself the parents who wouldn’t accept such things are another issue. We Iranians have this ridiculous shame which is a useless cliché in my opinion that causes us not to talk about this kind of matters in our families. Imagine if someone gets an STI or is pregnant without being married, she/he can’t even share and talk about it to get any help, so the situation gets constantly worse. I believe young people are scared of discussing these things and getting into trouble. (p9, M, 22, TH, LI)*

*They told us not to masturbate or you would go blind, meanwhile my eyes got weak, and I started wearing glasses, this made me feel guilty and I was getting bullied by the other kids for this reason and I had no way to prove them wrong. (p6, M, 25, AG, MI)*

*In Iran it is not legally supported for 2 people to love each other, have a relationship, get pregnant and then go and think about if they want to get married or not. (p4, M, 25, TH, LI)*

*There’s a fear of reputation and social credit as well. For example I have this friend who really wants to go to a gynecologist that her mom usually visits, because she knows she/he is a great doctor that her mom has chosen but she is worried that the doctor would call her mom and would tell her about her issues, she is scared of her mom’s reaction. (p4, M, 25, TH, LI)*

*The common belief is this fantasized picture of no one has sex before marriage whilst at least 90% of people have sex before getting married, to the extent that they take decisions to get married or not, solely based on their satisfaction with their sex life. But the common belief is that no way, 2 families are introduced together, and the groom sees the bride for the first time on that day and no one touches each other, and no one stays over. It’s hilarious the way society buries their head under sand, only not to see it all happening. (p4, M, 25, TH, LI)*

*In our culture, virginity is really important. I think it is the same with all Muslim countries. (p25, F, 25, R, LI)*

*I know girls who give in to any form of sexual relationship other than the vaginal intercourse only to protect their virginity, it’s a huge concern for so many people to the extent they put themselves in painful positions to please the guy they’re with but also to stay virgin. (p25, F, 25, R, LI)*

*Most young adults are stressed about this because it’s not a normal thing to lose your virginity before marriage or worse, getting pregnant. It is frowned upon. (p23, F, 20, AG, LI)*

*I believe here people are more concerned about what others might think of them not the disease itself. In my opinion you are 40% concerned about the disease and 60% concerned of others judgment. What others think of me is the main issue. That’s why I use protection. (p23, F, 20, AG, LI)*

*I am a lesbian, but I had to pretend I was straight for so many years. At first, I thought I have to be straight because it is the social norm. A friend of mine encouraged me to come out, she said you are a lesbian, you just haven’t figured it out. By social norms I mean people only find it normal if you are in a relationship with the opposite sex. It is a taboo to date the same sex although it is becoming more common. Even modern parents don’t accept it. (p22, F, 25, AG, LI)*

*In my opinion it's all about the limitations that parents create because they have issues with their daughters having a boyfriend, so the girls see it much easier to date girls. If you bring your girlfriend home, your mom wouldn’t find out. (p22, F, 25, AG, LI)*

*I believe it’s the cultural background. They are like we don’t have sex out of marriage and it’s a very sensitive issue. It is a taboo so you can’t tell people I had sex and because of that something has happened to me and now I need to go see a doctor. Or even nothing has happened, and I want to go see a doctor for my sexual health. This is not really common. Therefore I believe first is the fear that we have of the judgment we get and second is the taboo which hasn’t been broken yet and above all is the worry of what people might think and judge. (p18, F, 18, AG, HI)*

*I took part [in your study] because I know the majority wouldn’t accept to participate in such an interview easily because of the fear and taboos that exist within our society, so people don’t want to talk about their sexual life. They feel they should be embarrassed about their sexual relationships. (p12, F, 25, TH, MI)*

*One of the problems that we have is that until recent years, people from our generation couldn’t talk about sexual relationships with each other. I have 2 sisters that are born at the beginning of 80s and they never talked about their sexual relationship with each other or even with their friends, they see it as a taboo. When I started asking them about sexual stuff as their little sister, it was hard for them to answer and maybe a bit weird to talk about sex. Therefore this lack of communication resulted in my lack of information compared to other kids who are born after 1995-1996. (p12, F, 25, TH, MI)*

*It’s the fear of getting judged by the others. What others might think of me if they find out that I have sex out of marriage. I know so many guys that believe that their girlfriends are not decent and good people because they sleep with them. If she’s a good girl, why did she have sex with me? These backward, weird conservative way of thinking has roots in our male dominant society which put our women under so much pressure that she can’t find the courage even at the age of 30 to say I’m independent and this is my personal territory and not even my parents have the right to talk about my personal life. (p12, F, 25, TH, MI)*

*I don’t think I have any emotional support within my family or anyone I can talk to. So I’ll be double stressed in case I have an STI, that I have to tell them that I’m gay and I am ill. (p17, M, 19, TH, HI)*

*The thing that matters mostly in this case is that when it comes to sex others mostly your parents and people around are going to judge you. The worst feeling you can get is you are alone in this case. Whatever happens it’s on you, you have to take care of it by yourself, so that’s my biggest worry ever. It would be like it’s never going to be acceptable in their eye so it’s like for example parents don’t support you as a thief so it’s like you are a thief it’s not acceptable. So the worst feeling you can get is that you are alone in this. They are religious and also, they see this society they look at the majority of people who they think are not accepting sex out of marriage, so that’s why they judge you. You know if you think very deep through it you would be really scared inside. (p11, F, 25, TH, LI)*

*Another issue is that in families sexual relationships are a taboo, so they never talk about it with their children. Even the simplest stuff such as using condoms would not be taught to kids. When these young adults start getting into sexual relationships, they don’t have a clue about it, and they start to learn about it little by little by experimenting. (p12, F, 25, TH, MI)*

*Because they don't generally go to the doctor much, I don't know if it’s because of their families or the embarrassment they have to face within their families. (p3, F, 20, AG, HI)*

**10. Socioeconomic Sexual Health Inequalities in Tehran**

Mainly in response to question 10, 11 and 17 from the interview protocol.

10. Do you think sexual health education in Tehran needs to be extended or changed?

1. If so, how?

11. At what age do you think sexual health education should begin?

1. Should some topics be delivered at later developmental stages? What would you suggest?

17. Can you think of any reasons that young adults would avoid seeking sexual health knowledge and/or sexual healthcare in Tehran?

*In different parts of the city you can have different facilities, different shops, different parks, and different cinemas so it’s also the same with the gynecologists’ guess whatever you search for you can get, you can have access to different part of the city. It's going to be hard definitely but it's possible. (p11, F, 25, TH, LI)*

*For better doctors you have to go rich areas. (p11, F, 25, TH, LI)*

*And poor areas don't have much of a choice, both with doctors and contraceptives and condoms. (p1, F, 22, R, MI)*

*In deprived districts there is a lack of accessibility to certain sexual health facilities and also there are obvious financial issues in families living in these areas. (p6, M, AG, MI)*

*The situation is much better in affluent districts which means the accessibility decreases as you go to the more deprived areas. (p20, F, 23, AT, HI)*

*Certainly, the brands that are being sold here might have a high price and people who live in deprived areas can’t afford it so pharmacies in those areas won’t sell it in those areas. Therefore depending on which district you live in; you can find different stuff. (p23, F, 20, AG, LI)*

*I think it's more accessible in more affluent districts of Tehran and people would more comfortably purchase it. And as you go down to more deprived districts the accessibility decreases and even people are more ashamed or embarrassed to ask for Condoms. I mean it is not the same in different districts with different social classes. (p5, F, 25, AG, MI)*

*Expensive condoms are not available in deprived areas and if anyone wants to buy them, they would have to buy it from the affluent areas. (p6, M, 25, AG, MI)*

*Better and more expensive condoms can only be found in more affluent areas. (p8, F, 25, AG, HI)*

*The chance of finding high quality stuff with authentic brands in wealthier districts is way higher but it all depends on how much the person cares, if it is important to them, they would go to other districts and would pay for it, whatever the price. (p16, F, 25, AG, LI)*

*In affluent areas it's much easier to purchase. In these districts people have more purchasing power and are richer. Also there are more varieties in these districts. In deprived areas you can only find stuff to survive. I mean rich people take more care of themselves. Because their knowledge is much more. Therefore it is not accessible in every district, in deprived areas it is less accessible. (p24, F, 22, AG, LI)*

*I suppose you can find condoms everywhere but if it is the same brand, I’m not sure. I have never shopped in different areas. Even from time to time it’s different for example if I shop this month, I can find the brand if it goes to next month, I cannot find it. They cannot supply it from the same source. Maybe because everything is imported. I suppose some of them are from Thailand some of them are made in Iran also. (p11, F, 25, TH, LI)*

*In some districts of Tehran sex is still a taboo. The poor areas I mean. So what you can find here can’t be found there, because there is no demand for it. (p18, F, 18, AG, HI)*

**11. Gender power inequalities in sexual relationships**

Mainly in response to question 13 and 15 from the interview protocol.

13. How confident and in control do you think a typical 18–25-year-old Tehranian is in managing sexual relationships?

15. What contraception methods do you or your partner currently use or plan to use in your future relationships? Why?

*We don’t use condoms because he is not into it. (p24, F, 22, AG, LI)*

*I always strongly suggest using condoms although my boyfriend doesn’t like it at all, I don’t usually let it happen that we have unprotected sex more than 2 times in a month. (p19, F,24, TH,* *HI)*

*Even now if my partner doesn’t want to use condoms, I can’t tell him no. I think the problem is within me, I’m too shy and I also give in to my mental fantasies over my health. (p17, M, 19, TH, HI)*

*If he doesn’t want to use condoms I would accept because I don't want to annoy him. (p24, F, 22, AG,* *LI)*

*I’m comfortable with expressing opposition with the things that I don’t approve. (p15, F, 25, TH, LI)*

*Even if I get into a new relationship and I love my new partner very much I think I still can manage and control things that are really important to me. The things that are important to me are for example I never should be treated like a slave, I have to be respected, there needs to be mutual feelings involved and protection must be used. It is important that I’m in mood for having sex and they respect that. It’s happened that I said no, no matter how hard the person wanted it. (p19, F, 24, TH, HI)*

*I believe sexual relationships in Iran are used as a weapon. Men want sex and women want money and both sides use what they have to get want they want. Therefore nothing else matters to them, neither STIs nor sexual health knowledge. We have a society that doesn’t follow a certain way or form, it’s not completely conservative and traditional nor completely modern, but still marriage and being married have a great value and are important in our women’s lives. It is this thing that without it women don’t seem to have the value they should for the society. That’s why women try whatever they can to get married and to achieve this goal, they would do anything. Therefore when sex becomes something that gets you to money or whatever else you want, it wouldn’t matter if you are healthy, or you are actually enjoying it. You are just a tool in a system, that’s it. (p8,F,25,AG,* *HI)*

*I have this friend that her partner doesn’t like using condoms and asks her to do whatever she wants as a substitute contraception method, and she accepts because maybe she loves him or she wants sex with that person or she might want to look strong and cool in her partners eyes, so she puts herself at second place, something that so many women do in my opinion. In my opinion sex is related to confidence in so many ways. I mean it depends on how attractive or lovable you think you are. The stronger this feeling the more you can manage your relationships and the less you have this feeling, chances are you mess it up more and you can’t manage it properly. I think I’m not that confident yet to manage my relationships properly, for example if I really love someone and he wants to have sex without condoms I would accept because I don’t want to disappoint him although It scares the hell out of me, and I believe it’s totally wrong. As I said earlier, I believe many women would do it. (p5, F, 25, AG, MI)*

*There is this need to please guys in girls, and they tend to agree with whatever guys tell them, like not using condoms or having rough sex. I’ve seen this in my friends’ relationships. (p14, F, 25, R, LI)*

*It might happen that in the middle of sex would give in to whatever he wants in order to avoid ruining the pleasure of the moment. (p16, F, 25, AG, LI)*

*I should manage things for the sake of my own safety and health just the way I have managed it successfully so far and I should be able to carry it on. For instance using condoms, getting routinely checked by a doctor. (p12, F, 25, TH, MI)*

*In Iran boys control girls and they do whatever they want, and girls don’t have a say in it, the girls would be fooled and would say yes to anything like “No condoms?” Fine, “you don’t like it”? Fine. I’ve seen so many people like that. (p21, M, 25, AG, HI)*

**12. Recommendations for Improved Sexual Health Education and Services in Tehran**Mainly in response to question 21 from the interview protocol.

21. What would you top five recommendations for improved sexual health education and training in Tehran?

*In my opinion they should start with introducing the female and male’s sexual organs. Then they should talk about sexual health and preventive methods. (p1, F, 22, R, MI)*

*There should be mixed gender classes. It shouldn’t be formal but needs to be serious. They should let the participants share their opinions. (p1, F, 22, R, MI)*

*In my opinion schools should be mixed genders. They could then start sexual health education from junior high school like somewhere around ages 15-16. The age that sexual relationships might happen, there aren’t many people that start at younger ages. Puberty is a good age to start learning about sexual health. (p10, M, 24, TH, LI)*

*Short sessions would be enough. It should be mixed classes for both genders. It should be in small places with small amount of people, 10 people at max so that they can ask their questions comfortably. They should be able to have discussions. (p10, M, 24, TH, LI)*

*It should be done before you are 18…. To know their body, to know the limits, how much each person can get close to them, to learn about the opposite sex, the organs, how does it work? That’s it. (p11, F, 25, TH, LI)*

*It should be in a group, maybe groups of 10 and I think it should be done separately for each sex, so that its formal and no one makes fun. I would like to have discussions, maybe after the teaching part is over, we can actually have a discussion between 2 groups, girls and boys together and to get the feedback or maybe let them answer each other’s questions, that’s it. (p11, F, 25, TH, LI)*

*I think the age of 9 for girls and 11 for boys. Because girls start to get curious about sexual relationships at the age of 8 or 9 and they generally date and have sexual relationships with boys who are 1 or 2 years older than them. This is what I think because this is my own experience so when we start teaching them when they are 9 and 11 until they get to the age of 16 and 18, they have learned stuff that will be helpful to them. Girls’ periods usually start at the age of 9 or 10, so in an open-minded society we should teach them about the anatomy of their bodies so that they don’t get shocked when it all starts and make them prepared for it. It’s normal for kids to start noticing the opposite sex after the age of 10 or 11 so it would be better for them to know about it and be prepared so that if any sexual interaction happens for example at the age of 15, they know what they are doing and what are the risks and how they can protect themselves. (p12, F, 25, TH, MI)*

*It should be started from the beginning of elementary school with teaching about sexual organs, then they should carry it on with sexual health in middle school. (p13, F, 25, TH, LI)*

*Have specialists that people can trust such as health specialists and sexologists and ask them to educate people. Workshops and classes should be free so that everyone can comfortably participate without being concerned about the costs. People who can’t afford to pay for such classes are the main audience that need these classes and education. If we were in a country other than Iran, I would have said there is no difference but here in Iran classes need to be single sex. Because of the taboos, the embarrassment and the uncomfortable feeling that people have towards the opposite sex, they might not be able to ask their question comfortably. (p12, F, 25, TH, MI)*

*It should be mixed because if any questions come up both genders should know. There is nothing that should be taught differently for either sexes. That’s all. (p13, F, 25, TH, LI)*

*From the early years of elementary school they should teach kids about their sexual organs and after puberty which means around the ages of 12 to 13, they should start teaching about sexual health. (p14, F, 25, R, LI)*

*Some classes need to be mixed and some need to be single sex. Like some classes aimed at women’s health or men’s health should be held separately for each gender. The participants should be given brochures that are easy to read, interesting and engaging to take home with them. They should be allowed to discuss things and ask questions. (p14, F, 25, R, LI)*

*They should teach kids about sexual organs from the first year of elementary school. After the age of 12 they should start teaching sexual health. (p15, F, 25, TH, LI)*

*There should be maximum of 10 participants in each class, and they should be able to have discussions. The classes should be mixed gender. (p15, F, 25, TH, LI)*

*Teaching about sexual organs should start around the ages of 4 or 5 in order to avoid child molestation and sexual assaults not essentially for the sake of learning about sexual relationships. Sexual health and advice on sexual relationships should start at the ages of 12 or 13. (p16, F, 25, AG, LI)*

*Classes should be held with maximum 15 participants. The teaching material should be given to the participants in brochures or should be accessible on the internet. They should use visual stuff such as films or animation in order to make it interesting. The information should be put in the simplest way possible and easiest to comprehend. Some classes should be mixed and some not for example knowing about women’s health is not necessary for men, so it could be held for women only. (p16, F, 25, AG, LI)*

*In my opinion middle school is the time that kids start everything. They start becoming curious and want to try everything. I started to find out about this kind of things when I got into middle school before that I used to think I’m the result of my mom and dad praying. Praying at the same time for me and my brother and sister to get born. We are triplets. Anyway, I wouldn’t tell them about STIs because it would 100% makes them scared. I would explain about how these things happens scientifically. Like pregnancy and sexual orientation. I think knowing brings fear with itself. Then I would tell them about STIs and how they can use condoms to protect themselves. (p17, M, 19, TH, HI)*

*In my opinion everything needs to be told as soon as possible. Because there are so many kids who would start having sex at that time so it would be better for them to know it before it’s too late. It always better to know things as soon as possible. (p17, M, 19, TH, HI)*

*Some girls might not be comfortable to sit in a class with guys. I mean you should give them the option to choose if they want to participate in mixed gender classes or the single sex ones. There should always be a choice in this kind of situations. (p17, M, 19, TH, HI)*

*I believe the education must start at a very young age but it’s not necessary to open up everything for younger ages. I don’t believe we should separate genders from each other when everyone lives in peace and harmony together. We can tell them about their sexual organs. Kids in elementary school can get familiar with their body and what organs they have, how they look like and how they work. In middle school we should work on their sexual health and disease prevention. (p18, F, 18, AG, HI)*

*Families should stop treating this topic as a taboo and kids should get educated about it in schools. Schools should be mixed gender because children don’t start thinking about sexual relationships from the start, first we should fix the normal human relationships and emotional relationships. Here kids don’t have much interaction with the opposite gender on daily basis in schools so there isn’t a normal understanding and so when they get into the university which is mixed gender, the sexual side of human relationships get bold for them all of a sudden. They don’t have any emotional relationships with the girls, I am saying this because I’m a boy, but I am sure it is the same for the girls. (p10, M, 24, TH, LI)*

*I think health centers or places like that are a good place for holding classes. For example hold conventions in hospitals or in consulting institutes. (p18, F, 18, AG, HI)*

*From age 9. They’re not too young or too old. Just the right age to tell them things. First, I would tell them about the differences between boys’ and girls’ sexual organs. Then at the age of 10 or 11 I would teach them about how sexual relationships work and how they can protect themselves. (p19, F, 24, TH, HI)*

*The participants preferably should be from students or those who are about to get married. They need it the most. It should be mixed gender. Each class should have 20 to 30 participants so that they can ask their questions and learn from others. Discussions should be allowed. Teaching subjects should come with visual pictures and videos like power point or animation. (p19, F, 24, TH, HI)*

*Age 10. They could start with how pregnancy happens then as we grew older, they could teach us about contraception methods. The [educational material on] internet could also be beneficial. (p2, F, 18, AG, HI)*

*Flash cards are a good idea, for example for teaching about genitals. Classes should be mixed gender. Use of educational videos would also be nice. (p2, F, 18, AG, HI)*

*I would start it from year one of elementary school. Children should know that the body of a girl and a boy are different. Then in Middle school I would teach them about pregnancy and sexual health, however most of them would already know from talking to their friends. I started to find out about these stuff at the 4th year of elementary school (age 10) and as the time passed, I learned more. (p20, F, 23, AT, HI)*

*In my opinion it would be better for the classes to be mixed gender, so that we all benefit from it equally. In richer districts most people have the knowledge but if they hold the classes in city center locations, everyone can attend and improve their knowledge. The costs should be reasonable so that poor people can also attend and benefit. (p20, F, 23, AT, HI)*

*I would hold free classes because we love free stuff as a nation, just take a look at free food stalls! (p2, F, 18, AG, HI)*

*Conventions can be held for free; our people love free stuff. (p7, M, 21, TH, HI)*
